# Impact of serotonin transporter deficiency on parvalbumin- and neuropeptide Y-producing neurons of the lateral and basolateral amygdalar nuclei

**DOI:** 10.1007/s00702-026-03224-x

**Published:** 2026-08-19

**Authors:** Henning Schwert, Elif Salur, Mirjam Richard, Mara Pöllmann, Klaus-Peter Lesch, Esther Asan, Angelika Schmitt-Böhrer

**Affiliations:** 1 Institute of Anatomy and Cell Biology, University of Wuerzburg, Wuerzburg, Germany; 2 Department of Psychiatry, Psychosomatics and Psychotherapy, Center of Mental Health, University Hospital of Wuerzburg, Wuerzburg, Germany; 3 Division of Molecular Psychiatry, Center of Mental Health, University of Wuerzburg, Wuerzburg, Germany; 4 Department of Child and Adolescent Psychiatry, Psychosomatics and Psychotherapy, Center of Mental Health, University Hospital of Wuerzburg, Wuerzburg, Germany; 5 Department of Psychiatry and Neuropsychology, School of Mental Health and Neuroscience, Maastricht University, Maastricht, The Netherlands

**Keywords:** Basolateral amygdala, Inhibitory circuits, Monoaminergic systems, Anxiety, 5-HTT knockout mice

## Abstract

Hyperexcitability of lateral (La) and basolateral (BL) amygdalar nuclei (BLA) is a hallmark of anxiety-related disorders in humans. Excitation of BLA projection neurons (PN) is fine-tuned by inhibitory interneurons (INs), and monoaminergic afferents to the BLA modulate PN and IN activity. In the present study, BLA-neurons immunoreactive(ir) for parvalbumin (PV) or neuropeptide Y (NPY) and their interrelations with serotonergic and catecholaminergic afferents were analyzed in wildtype (WT) and in serotonin (5-HT) transporter knockout (5-HTT KO) mice, a mouse model for anxiety- and stress-related disorders. In both genotypes, PV- and NPY-ir neurons possess perisomatic appositions by serotonergic and tyrosine hydroxylase-ir afferents. Dual immunolabeling shows no colocalization of PV and NPY. Qualitative analysis of NPY/somatostatin(SOM) dual labeling in WT and 5-HTT KO mice documents colocalization of the peptides in neurons with predominantly fusiform somata, and single labeling for NPY in neurons with predominantly round somata. Quantification of PV- and NPY-ir neurons documents a reduction in numbers and densities of NPY-ir neurons of the BLA in 5-HTT KO mice while PV-ir neuron numbers and densities remain unchanged. Quantitative PCR shows increased expression of Npy receptor 2, Som receptor 4, and corticotropin releasing factor receptor 1 in the BLA of 5-HTT KO mice. mRNA for the peptides is unchanged, indicating that it may be NPY propeptide translation which is reduced in 5-HTT KO mice. Taken together, the results document an effect of life-long serotonin imbalance on the BLA NPY-system, which may contribute to increased anxiety-like behavior in 5-HTT KO mice.

**Supplementary Information:**

The online version contains supplementary material available at https://doi.org/10.1007/s00702-026-03224-x.

## Introduction

The amygdaloid complex plays a central role in the telencephalic processing of emotionally relevant, especially anxiety and fear-eliciting stimuli (LeDoux 2003; Pape and Pare 2010; Tovote et al. 2015). Amygdalar hyperactivity and/or enhanced stimulus responsivity are hallmarks of affective/anxiety disorders (Drevets et al. 2003; Brunetti et al. 2010; Brooks and Stein 2015; Shackman et al. 2016). Hyperexcitation or hyperexcitability of principal neurons (PN) in the basolateral nuclear complex (BNC) of the amygdala are thought to be a key feature of amygdalar hyperactivity (Almeida-Suhett et al. 2014; Tovote et al. 2015; Prager et al. 2016; Hinojosa et al. 2022). The BNC in rodents and primates consists of the lateral nucleus (La), the basolateral nucleus (BL; basal nucleus in primates) and the basomedial nucleus (BM; accessory basal nucleus in primates) (McDonald 2023). The BNC nuclei are derived from the ventral pallium (Garcia-Lopez et al. 2008; Garcia-Calero et al. 2020) and contain a majority of glutamatergic, pyramidal PNs and a minority of non-pyramidal neurons (NPN; McDonald 1984; Spampanato et al. 2011). Most NPNs are GABAergic interneurons (INs) which fine-tune the activity of PNs (Spampanato et al. 2011; Krabbe et al. 2018; Hajos 2021).

Information processing in the BNC is modulated by serotonergic (5-HT) and catecholaminergic, particularly dopaminergic (DA), afferents arising in raphe nuclei and the ventral tegmental area (VTA), respectively (Asan 1998; Asan et al. 2013; Bocchio et al. 2015, 2016a; McDonald 2023). Both for BNC PNs and for several groups of NPNs innervation by 5-HT and DA afferents has been documented in the rat (Asan 1998; Muller et al. 2007b, 2009; Bonn et al. 2013; McDonald 2023). Dysregulation of monoaminergic influence on BNC activity is thought to contribute to stress-related disorders, and therapeutic interventions directed at monoaminergic transmission modulate amygdala activity as shown by fMRI studies (Godlewska et al. 2016).

In addition to modulating brain circuits in the adult, 5-HT and DA play important roles in brain development and adult behavior (Yu et al. 2014; Suri et al. 2015), and considerable evidence links dysfunction of these systems to neurodevelopmental disorders (Lesch and Waider 2012; Money and Stanwood 2013). The DA system of the ventral tegmental area develops more slowly than the 5-HT system of raphe nuclei (Courtiol et al. 2021). Altered 5-HT transmission in early development has been suggested to lead to alterations in DA neuron/pathway function in the adult (Cunha et al. 2021¸ Menezes et al. 2021). The developmental role of the serotonergic system is of particular clinical relevance, since numerous genetic variants in 5-HT transmission molecules have been identified which influence individual differences in the stress response and in the risk for stress-related disorders (Holmes 2008). Thus, in humans and non-human primates, a functional polymorphism in the transcriptional control region of the 5-HT transporter (*5-HTT*, *SLC6A4*) gene, which leads to reduced production of 5-HTT in carriers of the short allele, has been shown to interact with early-life adversity to convey an increased risk for affective dysregulation (Lesch et al. 1996; Holmes 2008; Homberg et al. 2016). Overexpression or deficiency of the 5-HTT in mice affects amydalar responses to aversive cues, and impaired anxiety- and stress-related fear learning, memory and behavior. 5HTT-overexpressing mice are characterized by a low anxiety phenotype (Jennings et al. 2006) as well as altered amygdalar hemodynamic responses and theta oscillations, and display impaired fear learning and memory (Barkus et al. 2014); in contrast, 5-HTT knockout (KO) mice (Bengel et al. 1998) display increased (stress-related) anxiety-like and reduced aggressive behavior and exaggerated stress response with increased basal amygdalar activity (Holmes et al. 2002; Murphy and Lesch 2008; Line et al. 2011; Karabeg et al. 2013; Kolter et al. 2021). Additionally, 5-HTT KO mice exhibit faster discriminative fear learning associated with stronger theta frequency oscillations and greater hemodynamic changes in the amygdala in response to both the emotionally relevant cues and unconditioned foot-shock stimuli (Lima et al. 2019). The 5-HTT knockout (KO) mouse has been used as a mouse model for investigations into the neurobiological basis of anxiety- and stress-associated diseases (Holmes et al. 2003a, b; Kim et al. 2005; Holmes 2008). In these mice, PNs of the La and BL display increased dendritic spine density compared to wildtype (WT) mice (Nietzer et al. 2011). Increased spine density presumably represents a morphological correlate of PN hyperexcitation/hyperexcitability which may contribute to the increased anxiety phenotype of these mice (Wellman et al. 2007; Nietzer et al. 2011). PN hyperexcitation and dendritic plasticity may be caused by a dysregulated excitation/inhibition balance in BNC circuits, which in turn may be caused by alterations in the activity of inhibitory INs (Krabbe et al. 2018; Hajos 2021).

Morphological and neurochemical features of inhibitory BNC-NPNs have been analysed in detail in the La and in the anterior (magnocellular) and posterior (parvicellular) BL (together referred to in the present study as basolateral amygdala, BLA) in rodents. The BLA population of inhibitory neurons (approximately 15–20% of all neurons in the La, 20–25% of all neurons in the BL in rodents; Sah et al. 2003; Hajos 2021; Vereczki et al. 2021) comprises various subpopulations characterized by the separate, sometimes overlapping, expression of the calcium-binding proteins parvalbumin (PV), calbindin (CB), and calretinin (CR), and of neuropeptides such as cholecystokinin, vasoactive intestinal polypeptide, somatostatin (SOM) and neuropeptide Y (NPY) (Asan 1998; McDonald 1989; McDonald and Pearson 1989; Müller et al. 2007a; Pitkänen and Kemppainen 2002; Rainnie et al. 2006; Vereczki et al. 2016, 2021; Hajos 2021).

Particularly the PV-INs have been shown to exert important inhibitory control of BLA-PNs. PV-INs are GABAergic basket (appr. 80%) and axoaxonic (chandelier) cells which contact the somata and proximal dendrites or the axon initial segments of PNs, respectively (McDonald and Betette 2001; Muller et al. 2005, 2006; Bienvenu et al. 2012; Krabbe et al. 2018; Vereczki et al. 2016, 2021; Hajos 2021). Three morphological types of PV-INs have been described in the rat and monkey BNC (small and medium to large multipolar neurons, fusiform neurons, types I-III; Pitkänen and Amaral 1993; Kempainnen and Pitkänen 2000). By virtue of their somatic and axonal inhibitory innervation of PNs, PV-INs are powerful regulators of the excitation/inhibition balance which is critical for normal network function (Selten et al. 2018). Optogenetic studies implicated BLA-PV-INs in fear learning and fear extinction learning in mice (Wolff et al. 2014; Davis et al. 2017); a loss of BLA-PV-INs was found to be sufficient to increase avoidance behavior and impair fear learning (Colmers et al. 2025).

Of the peptides produced in NPNs, particularly NPY-mediated modulation of BLA circuits appears to play an important role in shaping fear responses. In rodents, effects of NPY in BNC nuclei in vivo include the induction of short- and long-term anxiolysis, and NPY in the BLA is thought to be crucially involved in the regulation of stress- and fear- or anxiety-related behavior (Kask et al. 2002; Yilmazer-Hanke et al. 2002, 2004; Gutman et al. 2008; Truitt et al. 2009; Cohen et al. 2012; Tasan et al. 2016; Bompolaki et al. 2024). Anxiolytic effects of NPY in the BLA appear to be mediated by activation of NPY receptor 1 (NPYR1) which in vitro has been shown to produce a direct hyperpolarization-induced inhibition of PNs. This can be countervailed by corticotropin releasing factor (CRF), a potently anxiogenic peptide (Giesbrecht et al. 2010). Somewhat counterintuitively, selective activation of the mainly presynaptic NPYR2 in the BLA has acutely anxiogenic effects (Sajdyk et al. 2002). In humans, a 30% decrease in NPY mRNA expression, due to a genetic polymorphism within the NPY promoter region, is associated with increased amygdala activity upon exposure to threat-related facial expressions (Zhou et al. 2008). NPY-producing BLA-NPNs comprise at least three neuron groups which can be distinguished by virtue of their colocalization of SOM: (1) a subpopulation of SOM-producing GABAergic INs which are reported to have elongated (fusiform/ovoid) somata (McDonald 1989; McDonald and Pearson 1989; Vereczki et al. 2021); (2) a small group of GABAergic projection neurons also coexpressing SOM possessing mostly fusiform somata (McDonald et al. 2012; McDonald and Zaric 2015; Bocchio et al. 2016b; Vereczki et al. 2021; Yamada et al. 2025), and (3) GABAergic neurogliaform cells (NGFC) which do not produce SOM and possess spherical somata (Vereczki et al. 2021; Ozsvar et al. 2024). SOM-INs, presumably including those that additionally produce NPY, are Martinotti cells forming synapses on distal dendritic segments and on spines of PNs (McDonald and Mascagni 2002; Muller et al. 2007a; Vereczki et al. 2021), and are thus in a position to influence processes of dendritic neuroplasticity. NGFCs are thought to release GABA predominantly via volume transmission, i.e. from axonal varicosities without a synaptic partner (Ozsvar et al. 2021; Ozsvar et al. 2024), and to act mainly at the dendritic compartment of PNs (Hajos 2021).

In the present study, we followed the hypothesis that dysregulation of inhibitory circuits formed by PV- and/or NPY-INs contributes to alterations in BLA excitation/inhibition balance in 5-HTT KO mice by analyzing the morphology, distribution and density of PV- and NPY-ir neurons in the La and BL in WT and 5-HTT KO mice. Additionally, we investigated interrelations of PV- or NPY-ir neurons in both genotypes with serotonergic afferents detected by 5-HTT- or 5-HT-immunoreactions (IRs; Asan et al. 2013) and with afferents detected by tyrosine hydroxylase(TH)-IRs, which have been shown to label mainly dopaminergic afferents in the rat and mouse (Asan 1993; Kalinowski et al. 2023). Finally, we analyzed mRNA expression levels of NPY and NPY receptors 1 and 2 (*Npyr1, -2*) and additionally of peptides and receptors which have been shown to be involved in mediation of anxiolytic or anxiogenic effects in the BLA, namely Somatostatin and Somatostatin receptors 2 and 4 (*Sstr2, -4*), CRF and CRF receptor 1 and 2 (*Crfr1, -2*), and serotonin receptors 1a, 2a and 2c (*5-HT1a, -2a, -2c*) (Giesbrecht et al. 2010; Scheich et al. 2016).

## Materials and methods

### Animals

Experiments were carried out on adult (> 10 weeks), male mice of a 5-HTT KO breeding line backcrossed into a C57BL/6J background for > 10 generations (Bengel et al. 1998). The animals were either homozygous for the wild-type (WT) allele or homozygous for the KO allele. Water and food were available *ad libitum*. All experiments were carried out according to the European Parliament and Council Directives (86/609/EEC and 2010/63/EU) and in accordance with the Guide for the Care and Use of Laboratory Animals of the Government of Lower Franconia (approval code 55.2-2531.01-93/12). The number of animals and the suffering induced was always kept as low as possible.

### Tissue preparation

For the single and dual immunolabeling studies mice were sacrificed by anaesthetic overdosing and transcardially perfused using 4% paraformaldehyde in 0.01 M phosphate buffered saline pH 7.4 (PBS). Brains were dissected, postfixed in the same fixative overnight, washed in PBS, cryoprotected in 10% and 20% sucrose in PBS, frozen in liquid nitrogen-cooled isopentane and stored at -80°. For vibratome sectioning, brains were slowly thawed to room temperature (RT), and coronal 35 μm-thick sections through the entire rostro-caudal extent of the amygdaloid complex were cut in PBS on a Vibratome (Leica, Wetzlar, Germany), collected in five series and used either immediately for immunolabeling or stored in cryoprotectant solution (Asan 1998) at -20 °C. One series of sections was mounted on Superfrost™ microscopic slides, and subjected to Gallocyanin staining (Heinsen et al. 2000). For cryostat sectioning, frozen brains were brought to ~-20 °C, cut at 18 μm thickness on a cryostat (Leica, Wetzlar, Germany) and thawed onto HistoBond^®^ adhesive microscopic slides (Marienfeld) additionally pre-coated with glycerin. Slides were stored at -80 °C until being used for immunolabeling.

For the gene expression study animals were sacrificed by cervical dislocation under deep anesthesia with isoflurane. Brains were then dissected and frozen in dry ice-cooled isopentane and stored at -80 °C until further processing.

### Immunolabeling

*Light microscopic (LM) immunoenzyme single labeling*. After thorough washing in 0.01 M phosphate-buffered saline pH 7.4 (PBS), free-floating vibratome sections were preincubated for two hours at room temperature in PBS with 1% Triton X-100 and 5% normal goat serum (NGS; blocking solution). Two adjacent series of vibratome sections were incubated in monoclonal mouse anti-PV-antibody (Millipore, #MAB1572; diluted 1:4000) and polyclonal rabbit anti-NPY-antibody (Sigma-Aldrich, #N9528; diluted 1:8000) in PBS with 0.5% Triton X-100 (Sigma), 1% NGS (incubation solution). Specificity of the antibodies was ascertained by the manufacturers; for the NPY antibody, absence of immunoreactivity has previously been shown in NPY KO mouse brain tissue (Theisen et al. 2018); additionally, the labeling patterns in our material was similar to published patterns of PV- and NPY-neuron distribution (Real et al. 2009; Nguyen et al. 2011; Vereczki et al. 2021; Ozsvar et al. 2024); For primary antibody incubation, 0.05%NaN_3_ was added to the incubation solution. Monoaminergic innervation was assessed in further section series using anti-tyrosine hydroxylase (TH; rabbit, polyclonal, Millipore, #AB152, 1:500), anti-5-HT (rabbit, Sigma, #S5545, or Immunostar, #20080, 1:2000 or 1:15000-1:40000), and anti-5-HTT (rabbit, polyclonal, Calbiochem, #PC177L, 1:500). Sections were incubated in primary antibodies for 48–72 h at 4 °C, washed in PBS, incubated overnight at 4 °C in biotinylated goat anti-mouse IgG or biotinylated goat anti-rabbit IgG secondary antibodies in incubation solution as required (Vector Laboratories, mouse: #BA-9200, 1:500; rabbit: #BA-1000, 1:500), washed again in PBS, incubated in avidin-biotin–horseradish-peroxidase complex (DAKO) prepared as decribed by the manufacturer in PBS for 2 h, and washed again in PBS. Visualization was carried out using the glucose-oxidase-diaminobenzidine (DAB) method described by Zaborszky and Heimer (1989). Immunolabeling on the majority of sections was intensified using nickel intensification (NiDAB; Asan 1997). Immunoreaction product development was checked by LM and stopped by repeated washes in PBS when maximum specific labeling was achieved. Sections were either washed in PBS and processed for dual immunolabeling as described below, or mounted onto Superfrost™ (Menzel, Braunschweig, Germany) microscopic slides, dried, dehydrated in a graded ethanol series and mounted in DePex (Serva, Heidelberg, Germany) from xylene. For *dual immunoenzyme labeling*, an established sequential incubation protocol was used (Eliava et al. 2003): Anti-TH, anti-5-HTT or anti-5-HT reacted sections developed using Ni-DAB were subjected to a second immunoreaction for detection of NPY or PV as described above, omitting the preincubation step. Immunolabeling was visualized using non-intensified DAB (Eliava et al. 2003).

*Single and dual fluorescence immunolabelings* were carried out by incubation of free-floating vibratome sections as described above with one primary antibody, or by simultaneous incubation of sections with two primary antibodies generated in different species, namely rabbit anti-5-HTT, mouse anti-PV, rabbit anti-NPY and rat anti-SOM (monoclonal, clone YC7, Sigma-Aldrich, #MAB354, 1:500). After washing in PBS, the sections were incubated with the appropriate fluorochrome-labeled secondary antibody or secondary antibody combination (Cy2- or Alexa488-labeled anti-mouse, anti-rat IgG or anti-rabbit IgG and Cy3- or Alexa555-labeled anti-rat or anti-rabbit IgG; Vector Laboratories or Invitrogen; dilution 1:200, 1:400 or 1:600). Secondary antibody incubations and subsequent washings in PBS were carried out as described above while keeping the sections in the dark. Mounted cryosections were washed in PBS, incubated in blocking solution for 2 h, incubated with anti-NPY and/or anti-SOM) in incubation solution (with 0.3% Triton-X-100) overnight at 4 °C in a humid chamber, washed again in PBS and incubated in secondary antibody solution for 2 h at room temperature (RT) and washed in PBS. In some sections, an antibody retrieval step (incubation in 10mM sodium citrate with 0.05% TWEEN 20, pH 6.0, for 20 min at 80 °C) was performed before preincubation with blocking solution. Fluorescent sections were counterstained using 4’,6-diamidino-2-phenylindole (DAPI). After washing in PBS, the free-floating sections were mounted on Superfrost microscopic slides. All fluorescent sections were covered with Fluoro-Gel (Electron-microscopic sciences, Hatfield, USA) and observed using a Zeiss Axioskop equipped with appropriate filter systems or the Compact Fluorescence Microscope BZ-X800 (Keyence, Neu-Isenburg, Germany).

*Controls* were performed for all single and dual immunolabeling experiments by omitting the primary antibodies for single labeling, by omitting either of the primary antibodies for dual fluorescence labeling, and by omitting the first and/or second primary antibody in sequential immunoenzyme labelings. Incubations with the respective secondary antibodies and, if applicable, Ni-DAB/DAB developments were performed according to the respective protocols. Additionally, individual sections of 5-HTT KO animals were incubated with the anti-5-HTT antibody. None of these preparations showed specific immunolabeling.

### Analysis of appositions between monoaminergic afferents and PV-ir and NPY-ir neurons

Perisomatic appositions were analyzed in dually DAB-immunolabeled amygdala sections by microscopically observing the sections using a 100x oil objective. Immunoreactive neurons containing a nucleus were selected and categorized according to their soma morphology (see below). The somata of labeled neurons were studied throughout all focal (z-axis) levels. Appositions of 5-HTT and TH-immunoreactive (-ir) fibers on PV or NPY-ir somata, in which no gap between the differently labeled structures was discernible microscopically, were recorded as appositions. For quantitative evaluations, perisomatic appositions were counted in dually labeled coronal sections at different interaural levels (-0.80 mm to -2.9 mm from Bregma; Franklin and Paxinos 1997) from 4 to 6 WT (5-HTT/PV-, 5-HTT/NPY-, TH/PV- and TH/NPY-labeled sections) and 4–6 5-HTT KO mice (TH/PV- and TH/NPY-labeled sections). Appositions were recorded separately for PV- and NPY-ir neurons in the different nuclei of the BLA. Mean apposition counts were similar in sections from individual animals for each category, indicating similar labeling quality of TH-ir or serotonergic afferents in the sections analyzed. Apposition counts were therefore pooled for mice of the respective genotypes in each category. Only categories with ≥ 4 neurons from ≥ 3 animals were used for statistical comparisons.

### Analysis of PV- and NPY-ir neuron numbers and densities

The number of neurons identified as PV- or NPY-positive in the La and BL was analyzed in six coronal immunoenzyme-reacted sections from 9 WT and from 8 5-HTT KO mice by an observer blinded for the respective genotype. The sections were spaced at least 140 μm apart, with the two rostral-most sections (between − 0.80 and − 1.5 mm from bregma) representing anterior amygdaloid levels, two middle sections (between − 1.5 and − 2.2 mm) representing intermediate, and two caudal sections (between − 2.2 and − 2.9 mm) representing posterior levels of the amygdala. The La and the anterior and posterior subdivisions of the BL (BLa, BLp) were delineated in each section according to the “Mouse Brain in Stereotaxic Coordinates” (Franklin and Paxinos 1997). Delineation in difficult cases was supported by comparison with Gallocyanin-stained adjacent sections, and neuron numbers were recorded only if delineation of nuclei/subnuclei was unequivocally possible. Immunolabeled neuron numbers, positions within the La/BL and borders of the respective nuclei were recorded using the “Stereo Investigator” software (version 10.52) and the Neurolucida explorer (version 10.52; MicroBrightfield Europe B.V., the Netherlands). Neuron density in the different nuclei, subnuclei and rostro-caudal regions was calculated by dividing the number of neurons counted by the respective amygdalar nuclear volume analyzed (area analyzed x section thickness) and depicted as number/10^6^ µm^3^.

### Gene expression analyses

Gene expression analyses were carried out on ten male 5-HTT WT and eight male 5-HTT KO mice. For Laser capture microdissection, Brains were sectioned at 20 μm in a Leica CM1950 freezing microtome at -20 °C and the tissue sections were mounted on polyethylene naphthalate (PEN) membrane slides 2.0 μm (Leica, Wetzlar, Germany). Mounted tissue sections were fixed in -20 °C cold 75% Ethanol (diluted in DEPC-treated water) for 2 min, stained with 0.25% cresyl violet and then processed through an ascending ethanol series (75%, 90%, 100% ethanol for 20 s each) for decoloring. After an additional incubation for 1 min in 100% ethanol, the slides were air dried in 50 ml tubes with Silica Gel Orange (Roth, Karlsruhe, Germany), and the tissue was dissected using a Leica LMD6 laser microdissection system, which allowed the precise extraction of the La/BL (-0,94 to -1,82 from bregma).

RNA was extracted from the dissected tissue using the miRNeasy Micro Kit (Qiagen, Hilden, Germany) and stored at -80 °C until further use. The iScript cDNA Synthesis Kit from Bio-Rad was utilized for Reverse transcription of the RNA into cDNA. As the LMD only results in low amounts of harvested tissue, extracted RNA and subsequent cDNA, an unbiased, target-specific preamplification of cDNA using the 2x SsoAdvanced PreAmp Supermix (Bio-Rad) was necessary. Primer sequences to detect the expression levels of genes related to NPY, SOM, CRF, and the serotonergic system required for this pre-amplification step as well as for the quantitative real-time PCR (qPCR), were selected using the NCBI BLAST tool (Online Resource 1) and purchased from Sigma-Aldrich (St. Louis, MO, USA). To perform the qPCR reactions, the pre-amplified cDNA stock solution was further diluted using water to a 1:20 working solution. Per sample, 0.5 µl forward and reverse primer (5 µM each) were mixed with 5 µl SYBR™ Select Master Mix (Thermo Fisher Scientific, Waltham, MA, USA) and 4 µl cDNA to a total volume of 10 µl. A 384-well plate in a CFX384 real-time PCR detection system (Bio-Rad) was used for the reactions. LinRegPCR program (version 2017) (Ruijter et al. 2009) was used to calculate mean PCR efficiencies. Evaluation of expression, normalization and calculation of relative gene expression results were obtained with qBase+ software (Biogazelle, Ghent, Belgium).

### Statistics

Both data derived from immunolabeled sections as well as gene expression data were analyzed using SPSS Statistics version 26 (IBM Corporation, New York, USA), and GraphPad Prism software version 10 (GraphPad Software, La Jolla, California, USA) was used for data graphing.

For quantitative analyses of PV and NPY-ir neurons, data derived from immunolabeled sections (volume of the respective amygdala subregion, number of immunopositive cells, density of immunopositive cells) were analyzed by a mixed two-way analysis of variance with all amygdalar subregions under investigation (BL vs. La, BLa vs. BLp, BLA as well as BL and La: rostral vs. intermediate vs. caudal) serving as the within-subjects factor and the two different *5-Htt* genotypes serving as the between-subjects factor, followed by Bonferroni’s post hoc analysis whenever appropriate.

For statistical analysis of the number of appositions and the gene expression data sets a Levene test for homogeneity of variances and a Shapiro-Wilks test for normal distribution were first performed. Since the Shapiro-Wilks test indicated that the apposition data pooled from all animals in a group were not normally distributed, a pairwise comparison of the number of appositions was performed using the nonparametric Mann-Whitney U test. In the statistical analysis of the gene expression datasets, a t-test was performed provided that the variance distributions were not significant (*p* > .05) and the samples were normally distributed. If the requirements for a t-test for independent samples were not met, the Mann-Whitney U-test was used for pairwise comparisons.

Statistical significance was set at *p* < .05. Significance levels are indicated (if not otherwise indicated in the graph legends) as * *p* < .05, ** *p* < .01, *** *p* < .001.

## Results

### Distribution and morphology of PV- and NPY-ir neurons in WT and 5-HTT KO mouse BLA

#### PV-ir neurons

**Fig. 1:**
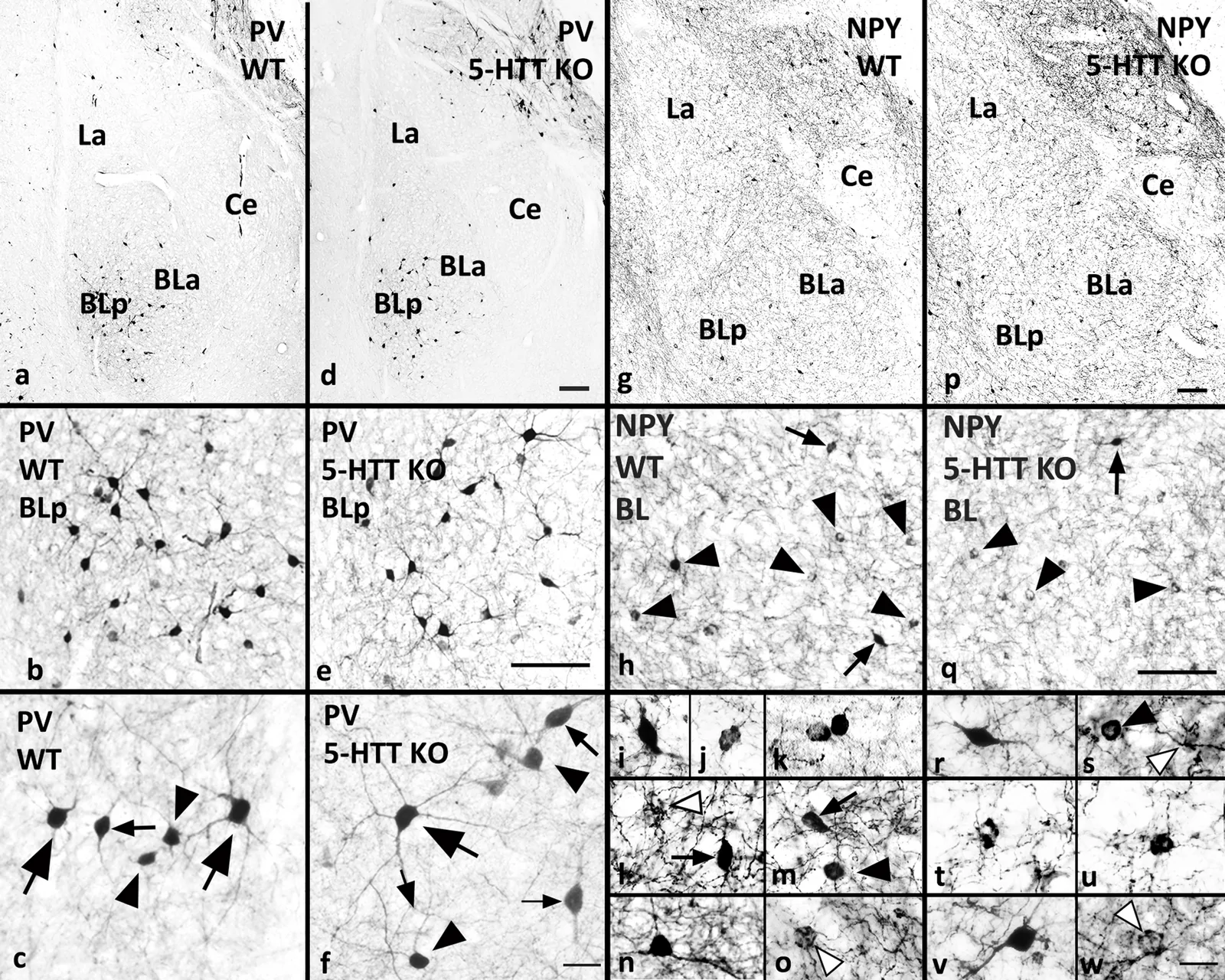
(**a-f**) Immunoenzyme labeling for PV in the WT (a-c) and 5-HTT KO mouse BLA (d-f). In mice of both genotypes, PV-ir neurons are densely clustered in the posterior basolateral nucleus (BLp; b, e), less dense in the anterior BL (BLa), and least dense in the lateral nucleus (La). (**c, f**) Type I (arrowheads), II (large arrows) and III (small arrows) PV-ir neurons in the WT and 5-HTT KO mouse. (**g-w**) Immunoenzyme labeling for NPY in the WT (g-o) and 5-HTT KO mouse (p-w). (**g, h** and **p, q**) NPY-ir neurons are scarcer but more evenly distributed than PV-ir neurons in the BLA of both genotypes. In (h) and (q), arrows point to fusiform, arrowheads to round neurons. (**i, j, l, m,** and **r, t**) Neurons with fusiform somata (i, j, r, t and arrows in l, m). Note the inhomogenous cytoplasmic staining in some fusiform neurons shown in (j) and (t). (**n, v**) Neurons with ovoid or polygonal somata. (**k, m** and **s, u**) Round NPY-ir neurons (k, u and black arrowheads in m, s) show different labeling intensities. (**l, o,** and **s, w**) Occasionally, NPY-ir elements appear to densely surround an apparently unlabeled neuronal soma (white arrowheads). Ce: central amygdaloid nucleus. Bars: 100 µm in d for a, d, in p for g, p, in e for b,e and in q for h, q; 20 µm in f for c, f and in w for i-o and r-w

In both WT and 5-HTT KO mice, immunoenzyme labeling detects the highest density of *PV-ir neuronal somata* in the BLp, lower density in the BLa, lowest in the La (Fig. 1a-f). In analogy to the morphological types of BLA PV-neurons described in the rat (Kemppainen and Pitkänen 2000), most PV-ir neurons in both genotypes possess three to five dendrites and round to oval somata of small size (type I PV-INs) or comparatively large size (type II PV-INs). Additionally, some PV-ir neurons (type III) possess fusiform somata and dendrites originating mainly from the two cell poles (Fig. 1c, f). The reaction product is homogeneously distributed in somata and proximal dendrites. The intermediate and distal dendrites are not commonly labeled. The proximal dendrites of all neurons display smooth contours.

#### NPY-ir neurons

In both genotypes, NPY-ir neurons in immunoenzyme-labeled sections are less frequent and appear more homogeneously distributed throughout the La and BL than PV-ir neurons (Fig. 1g, h, p, q). The following description applies to NPY-ir neurons in the BLA of both the WT and 5-HTT KO-mice. BLA-NPY-ir neurons can be divided into two groups based on soma shape: neurons with elongated, most often fusiform or oval, occasionally polygonal somata (Fig. 1h, i, j, l, m, n, q, r, t, v) of various sizes (in the following text summarily referred to as “fusiform neurons”) and neurons with spherical somata (“round neurons”; Fig. 1h, k, m, q, s, u). The immunoreaction product is homogeneously distributed throughout the somatic and proximal dendritic cytoplasm in most fusiform neurons (Fig. 1i, l, n, r, v). Occasionally, fusiform neurons are observed in which the immunoreaction product is inhomogeneously distributed (“cytoplasmic streets” and “spots”), particularly in the smaller ones of this group (Fig. 1j, t). Immunolabeled proximal dendrites are narrow and smooth. Most round neurons possess comparatively small somata with immunoreaction product often unevenly distributed in the cytoplasm (e.g. located predominantly on one side of the soma, or in cytoplasmic spots; Figs. 1k and 3d-f). Most NPY-ir fiber plexus are constituted by narrow fibers with numerous small varicosities. Occasionally, NPY-ir varicose elements are observed in the periphery of apparently non-immunoreactive somata (Fig. 1l, o, s, w). In these cases, it is often possible to trace the varicose structures to NPY-ir fibers when viewed through different focus levels. However, in some neurons it is difficult to discern inhomogeneous perikaryal labeling from varicose fibers running alongside the soma.

**Fig. 2:**
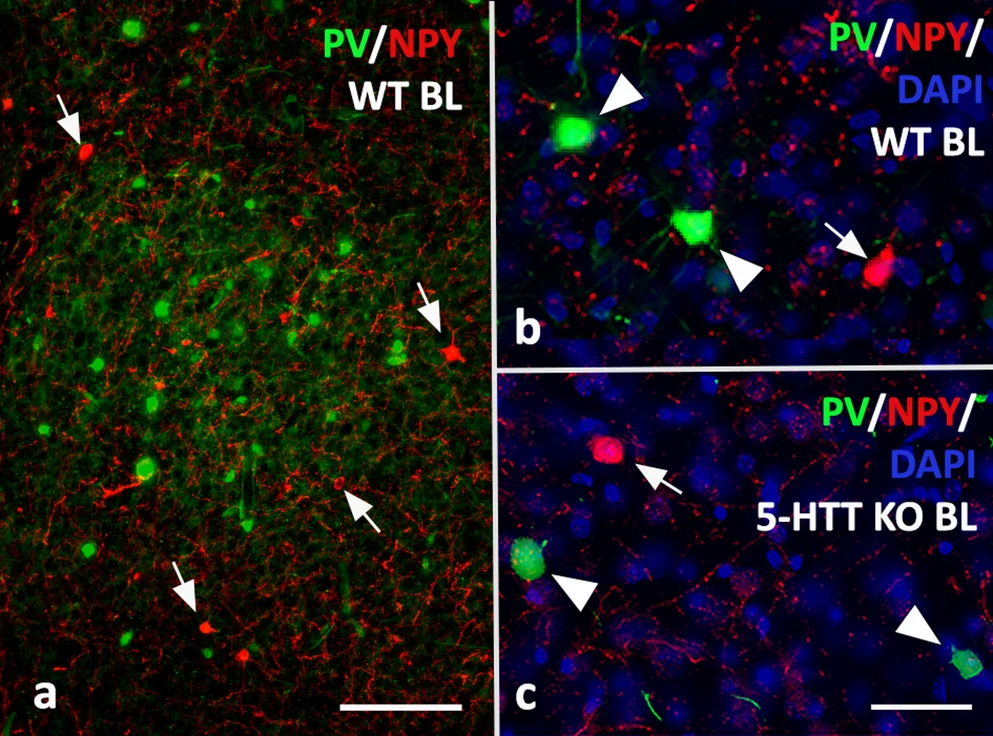
(**a**) Dual immunofluorescence labeling for PV (green) and NPY (red) at low magnification and (**b, c**) at higher magnification with DAPI shows localization of PV and NPY in separate populations of neurons in the BL of WT and 5-HTT KO mice (arrows: NPY-ir neurons, arrowheads in b, c: PV-ir neurons). Bar in a: 100 µm, in c for b, c: 50 µm

### Colocalization of PV- or SOM-immunoreactivity in NPY-ir BLA neurons in WT and 5-HTT KO mice

**Fig. 3:**
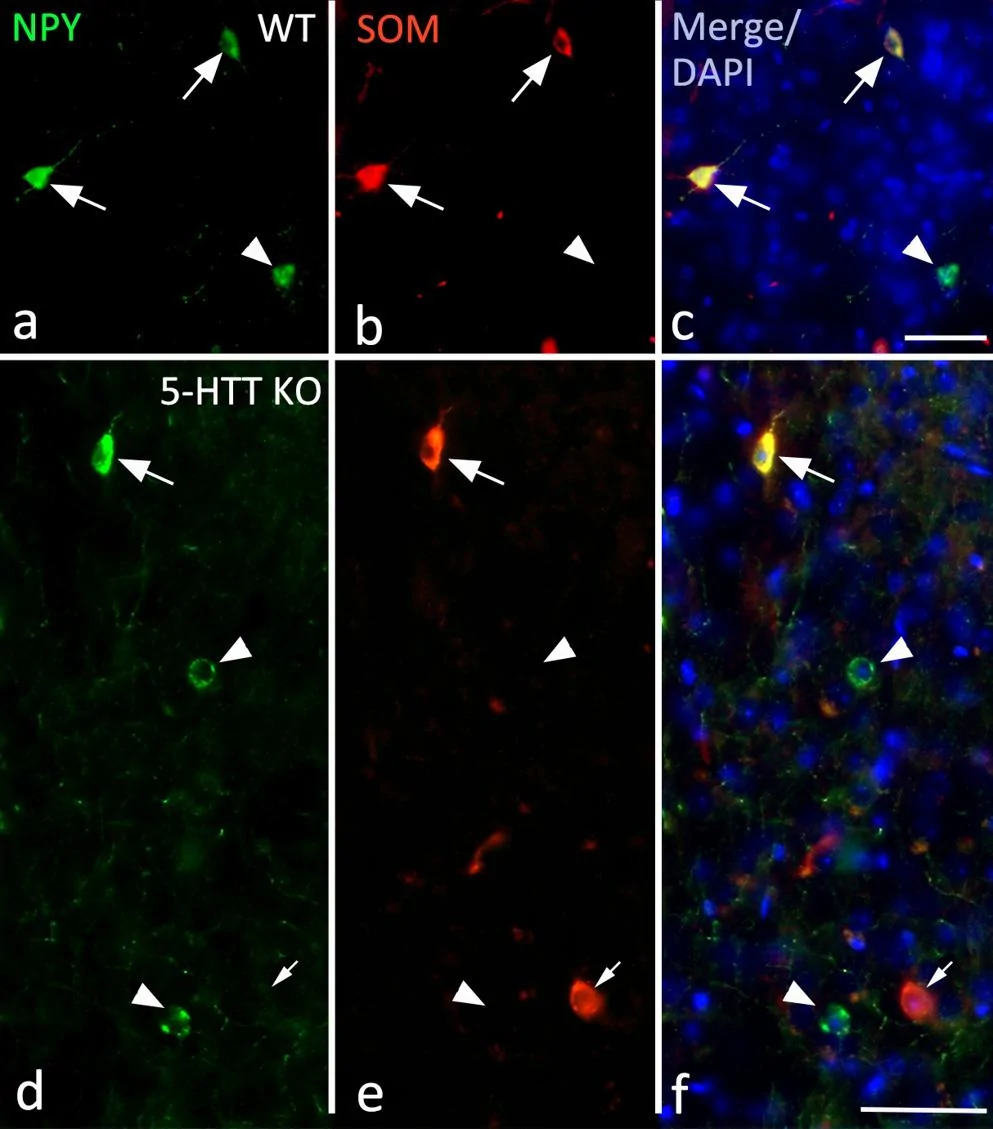
Dual immunofluorescence labeling for NPY and SOM in the BLA of WT (**a-c**) and 5-HTT KO mice (**d-f**) shows double-labeled neurons (large arrows) as well as SOM-single labeled (small arrows) and NPY-single labeled neurons (arrowheads). Single NPY-ir neurons often possess small round somata, double-labeled and single SOM-ir neuronal somata display elongated (fusiform, ovoid or polygonal) shape. Vibratome sections, Bars = 50 µm

**Fig. 4:**
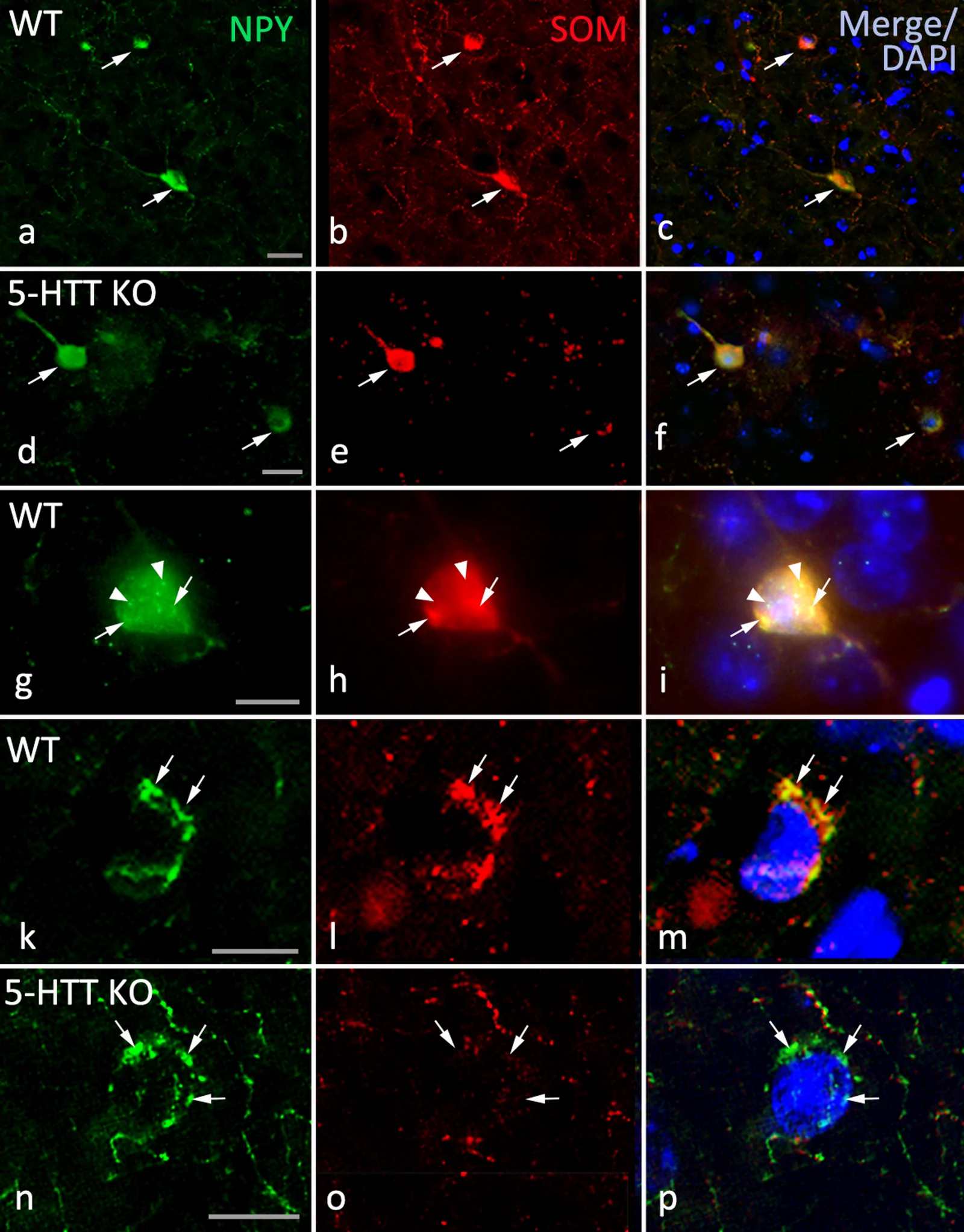
Dual Immunolabeling for NPY und SOM in the BL of WT (**a-c, g-m**) and 5-HTT KO mice (**d-f, n-p**). (a-f) Immunolabeling intensities for SOM and NPY vary between double-labeled neurons (arrows) both in WT (a-c) and in 5-HTT KO mouse BLA (d-f). (**g-i**) In this polygonal soma, SOM and NPY immunofluorescences are homogeneously distributed in the perikaryal cytoplasm and additionally concentrated in overlapping compartments (arrows). Intense NPY-IR appears localized in additional compartments (arrowheads). (**k-m**) SOM- and NPY-IR are localized in overlapping perikaryal compartments (arrows). (**n-p**) Single NPY-IR is found in varicose structures surrounding neuronal nuclei. Differentiation between labeling in perikaryal compartments as opposed to NPY-ir varicose axons surrounding the perikaryon (cf. Fig. 1 h, i) is not always possible. a-f and k-p: cryostat sections, g-i: vibratome section. Bar in a, d: 20 µm, in g, k, n: 10 µm

In both genotypes, PV/NPY-double-labeled cells are not observed in the BLA (Fig. 2). In contrast, NPY/SOM-double-labeled neurons in addition to SOM- or NPY-single-labeled neurons are found in La and BL of WT and 5-HTT KO BLA (Figs. 3 and 4). SOM/NPY double labeled somata in the BLA nuclei are often elongated (fusiform, ovoid; Fig. 3a-f). Additionally, occasionally SOM/NPY double labeled neurons with relatively large, fusiform to polygonal somata and some double-labeled neurons with round somata of varying size are observed (Figs. 3a-c and 4a-f). The relative immunofluorescence intensity for the two peptides differs between double-labeled neurons (Fig. 4a-f). In some double-labeled neurons, immunolabeling for NPY and SOM is homogeneous throughout neuronal perikarya and extends occasionally also into dendrites (Fig. 4a-f). In the perikaryal cytoplasm, IR for NPY and SOM is often localized in specific, largely but not in all cases completely overlapping compartments (Fig. 4g-m). NPY-ir single-labeled neurons usually possess small round somata with NPY-IR concentrated in cytoplasmic streets or spots (Fig. 3). In some cases, particularly in cryostat sections, it is difficult to discern perikaryal labeling of small neurons from NPY-ir fiber varicosities surrounding neuronal somata (Fig. 4n-p, cf. Figure 1o, w). Attempts to quantify the proportion of the different types of NPY-ir neurons in double labeled sections from WT and 5-HTT KO mice yielded heterogeneous results (data not shown), possibly because the fluorescence of labeled neurons was sometimes very faint with fluorescence bleaching additionally lowering the detectability of specific labeling. Therefore, the results were deemed unreliable and are not reported here.

### Interrelations between monoaminergic innervation and PV- and NPY-producing BLA neurons in WT and 5-HTT KO mice

**Fig. 5:**
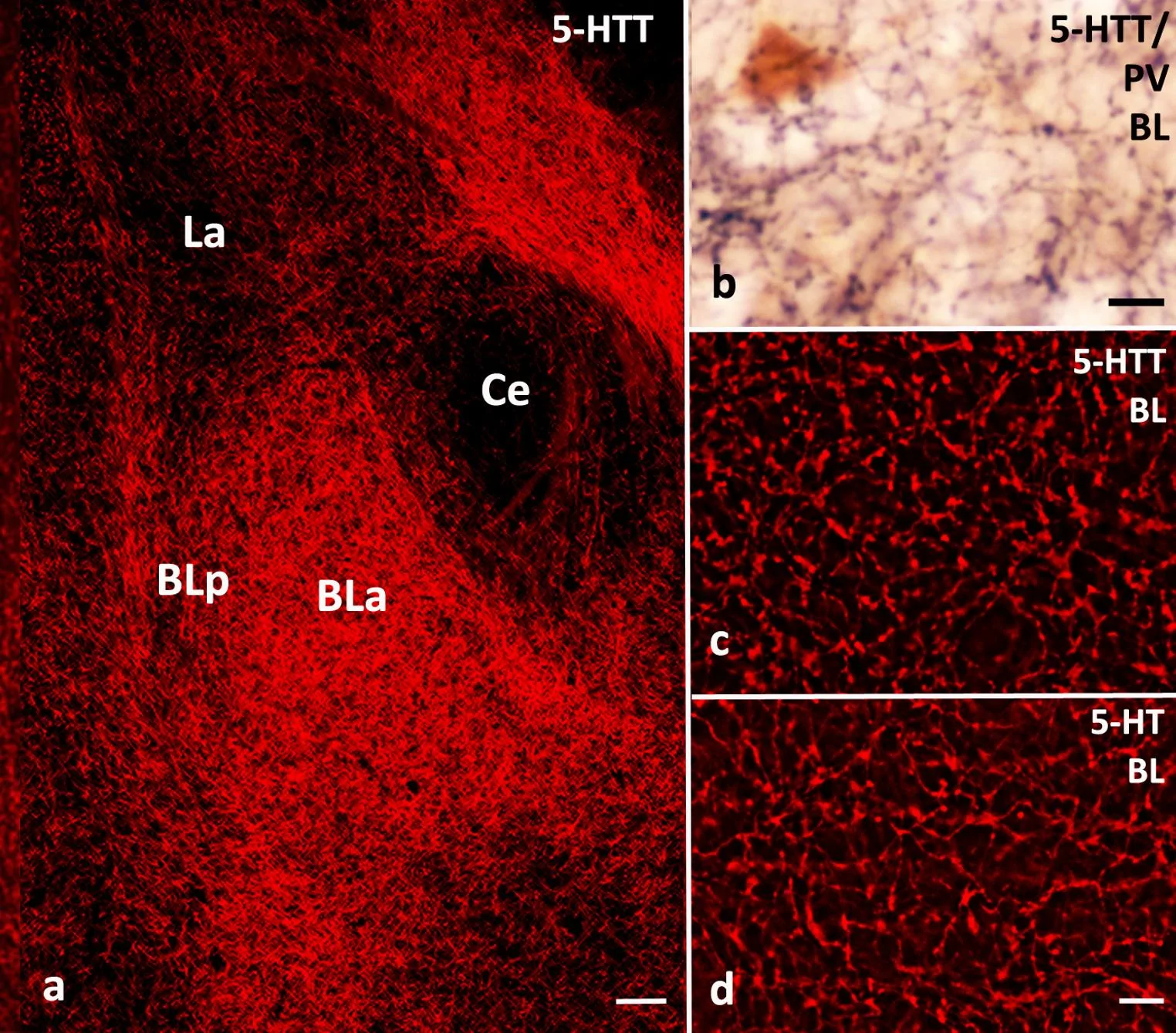
Serotonergic fibers in the WT mouse. (**a**) 5-HTT immunofluorescence shows dense serotonergic fiber plexus in the BLa, somewhat less dense in the BLp and least dense in the La. Ce: central amygdaloid nucleus. (**b**) 5-HTT-ir fiber density in dual immunoenzyme labeling for 5-HTT (black)/PV (brown) is comparable to density in single 5-HTT labelings. (**c, d**) Immunoenzyme-labeled 5-HTT and 5-HT fibers are shown after digital conversion into false colors. In these images, the density of 5-HT-ir fibers (d) is similar to that of 5-HTT-ir fibers (c). Bar in a: 100 µm, in b: 10 µm, in d for c and d: 10 µm

**Fig. 6:**
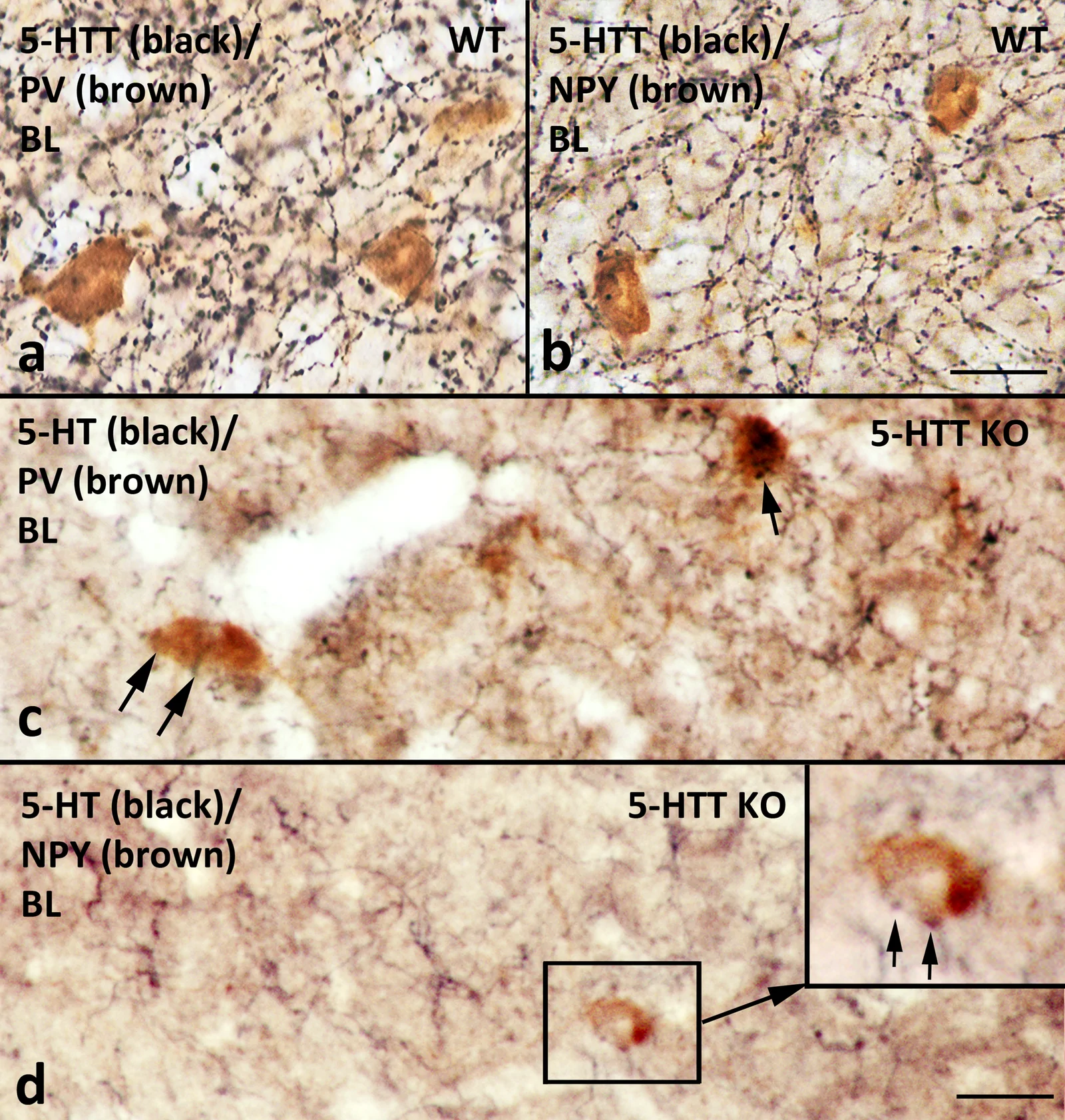
Dual immunoreactions showing perisomatically localized serotonergic fibers labeled by 5-HTT antibodies in the WT (a, b) and by 5-HT antibodies in 5-HTT KO mice (**c, d,** arrows) on PV (a,c) and NPY-ir neurons (b,d) in the BL. Bars in b for a, b and d for c, d: 20 µm

5-HTT immunoreactions in WT result in intra- and interindividually reproducible labeling of dense serotonergic fiber plexus in the BLA (Figs. 5a-c and 6a and b). 5-HTT-ir fiber density in 5-HTT/PV- (Figs. 5b and 6a) and 5-HTT/NPY-dual immunolabeled sections (Fig. 6b) is as high as in single labelings (Fig. 5a, c). 5-HTT-ir axons display numerous irregular varicosities (Figs. 5b and c and 6a and b). 5-HTT fiber density is higher in the BL than in the La (Fig. 5a) and appears slightly higher in the BLa than in the BLp. In contrast to 5-HTT immunoreactions, 5-HT immunoreaction results vary considerably between animals. In 5-HT labeled sections of WT mice showing a density of serotonergic fibers comparable to that of 5-HTT labelings in the respective (sub)nucleus (Fig. 5c, d), 5-HT-ir fibers are observed to be fine and tortuous with pleomorphic varicosities (Fig. 5d).

*Perisomatic appositions of serotonergic afferents on PV- and NPY-ir BLA neurons.* Numerous axons running close to PV- or NPY-ir somata are observed in 5-HTT/PV and 5-HTT/NPY immunoreactions in the WT (Figs. 5b and 6a and b). In 5-HTT KO mice, PV- and NPY-ir neurons display perisomatically localized 5-HT-ir fibers in both the BL and La (Fig. 6c-d). However, the interindividual variability of the 5-HT immunoreaction quality precludes quantitative analyses of 5-HT/PV and 5-HT/NPY dual labeled material. Such analyses were therefore only carried out for appositions of 5-HTT-ir fibers in the WT mice (Table 1).

**Fig. 7:**
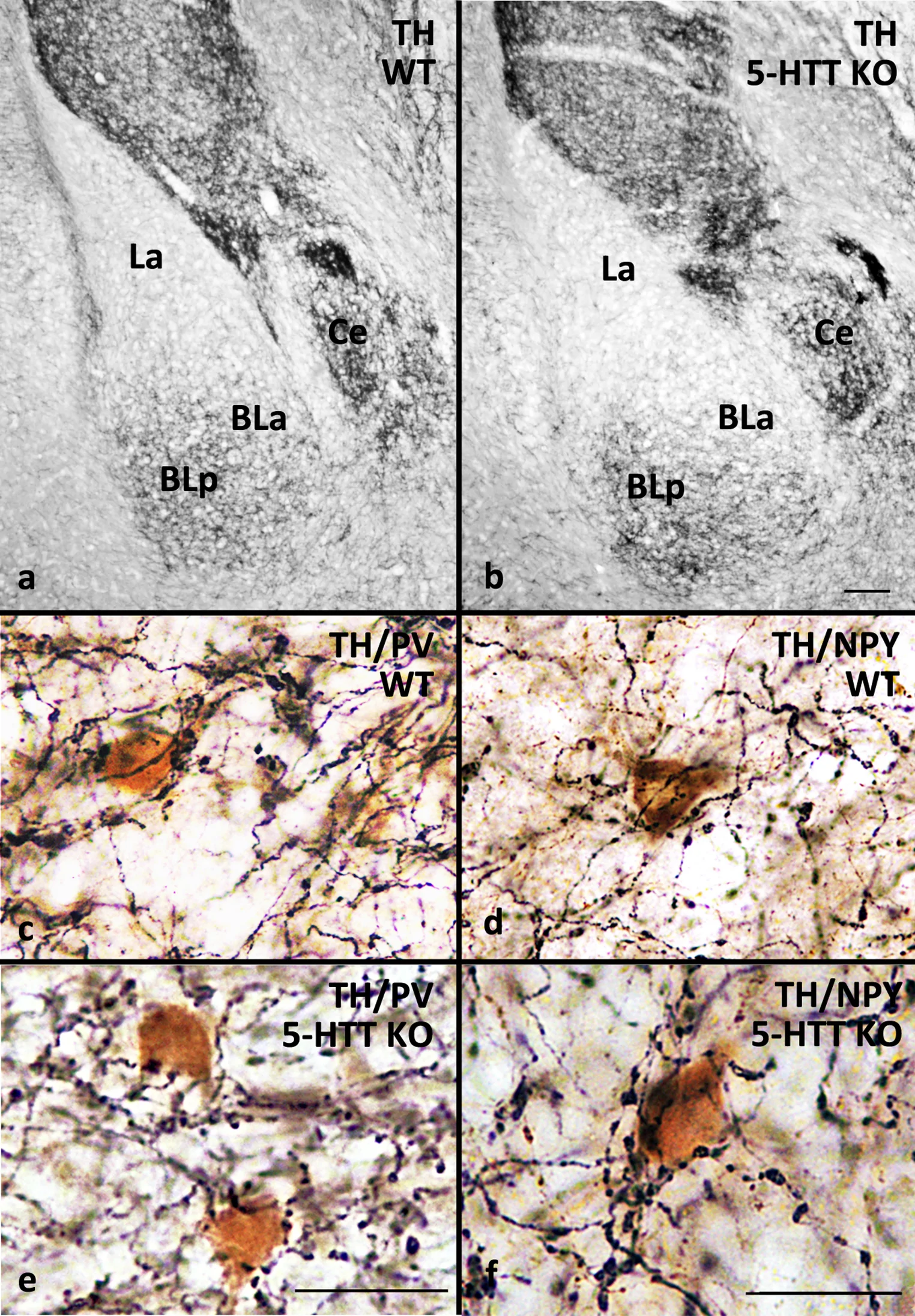
TH-ir fibers are heterogeneously distributed in the BLA of WT (**a**) and 5-HTT KO mice (**b**), with highest fiber density in the posterior basolateral nucleus (BLp), less dense plexus in the anterior BL (BLa), and scarce plexus in the lateral nucleus (La). Narrow varicose TH-ir fibers run close to the somata of PV-ir (**c, e**) and NPY-ir neurons (**d, f**) in the BL of both WT (**c, d**) and 5-HTT KO mice (**e, f**). Ce: central amygdaloid nucleus. Bar in b for a, b: 100 µm; Bars in e, f for c-f 25 µm

*BLA innervation by TH-ir fibers.* Highest density of TH-ir fibers in the WT is found in the BLp, less dense plexus in the BLa, scarce plexus in the La (Fig. 7a). The terminal fibers are thin and smooth with narrow and irregularly arranged varicosities (Fig. 7c, d). Distribution, density and morphology of TH-ir afferents in the 5-HTT KO mice (Fig. 7b, e, f) closely resemble those found in the WT. Numerous varicose TH-ir afferents run close to the somata of PV- and NPY-ir neurons in BLA of both WT and 5-HTT KO mice (Fig. 7c-f; Table 1).

### Quantitative assessment of perisomatic appositions of 5-HTT- and TH-ir afferents on PV and NPY-ir BLA neurons (Table 1)

*Appositions of 5-HTT-ir fibers on WT PV-ir neurons.* On average, PV-neurons in the BLA receive at least three perisomatic appositions by serotonergic afferents. Appositions on PV-ir neurons are significantly more numerous in the BL than in the La (*p* ≤ .001), with apposition frequency in the La amounting to about 79% of that observed in the BL.

*Appositions of 5-HTT-ir fibers on WT NPY-ir neurons.* NPY-ir neurons in the BL and La receive, on average, four and three perisomatic appositions, respectively, by 5-HTT-ir afferents. Appositions in the BL are significantly more frequent than in the La (*p* ≤ .001; apposition frequency 72–78% for different NPY-ir neuron types in La compared to BL). No statistically significant difference in apposition counts is found between round and fusiform neurons.

*Appositions of TH-ir fibers on PV-ir neurons.* In both genotypes, *PV-ir neurons* in the BL have the highest number of perisomatic appositions. Each randomly selected PV-ir neuron possesses more than three appositions. In the La, perisomatic appositions on randomly selected PV-ir neurons are significantly less frequent than in the BL: only 58% and 69% of the analyzed neurons possess perisomatic appositions by TH-ir fibers in the WT and 5-HTT KO, respectively (data not shown). Mean apposition frequency in the La thus amounts to 28 and 27% of that in the BL for PV-ir neurons in the WT and the 5-HTT KO (*p* ≤ .001, Table 1).

*Appositions of TH-ir fibers on NPY-ir neurons*. In both WT and 5-HTT KO BL, each randomly selected NPY-ir neuron displays perisomatic appositions. In the La, only 47.5% and 39% of analyzed NPY-ir neurons show perisomatic appositions by TH-ir fibers in the WT and 5-HTT KO animals, respectively (data not shown). Mean apposition frequency in the La thus amounts to only about 18% (range: 18–19%, *p* ≤ .001, Table 1) of that in the BL in WT and 16% (range 13–19%, *p* = .001 to ≤ 0.001) in 5-HTT KO La. Differences in apposition frequencies between the morphological subtypes are not observed in WT. In 5-HTT KO BL, round NPY-ir neurons possess less appositions than fusiform neurons (*p* = .012), and less than their counterpart in the WT (*p* = .03).

**Table 1:** Appositions of 5-HTT-ir and of TH-ir afferents on randomly selected PV- and NPY-ir neurons in the BL and La of WT and 5-HTT KO mice

Neuron type	Appositions of 5-HTT-ir fibers in WT		Appositions of TH-ir fibers in WT		Appositions of TH-ir fibers in 5-HTT KO	
	BL	La	BL	La	BL	La
PV all	**4.28** ± 0.13	**3.38** ^***^ ± 0.18	**3.77** ± 0.20	**1.07** ^***^ ± 0.18	**3.82** ± 0.10	**1.06** ^***^ ± 0.13
NPY all	**4.00** ± 0.15	**3.00** ^***^ ± 0.15	**3.35** ± 0.16	**0.61** ^***^ ± 0.11	**3.04** ± 0.16	**0.50** ^***^ ± 0.17
NPY round	**4.00** ± 0.20	**3.13** ^**^ ± 0.13	**3.41** ± 0.21	**0.61** ^***^ ± 0.15	**2.69** ^**§**^ ± 0.21	**0.50** ^**^ ± 0.34
NPY fusiform	**4.00** ± 0.19	**2.90** ^**^ ± 0.26	**3**,**22** ± 0.28	**0**,62^***^ ± 0.18	**3.56** ^**+**^ ± 0.18	**0.50** ^***^ ± 0.19

Data represent mean ± SEM. Comparison BL/La: ^*^p< .05; ^**^p< .01; ^***^ *p*< .001; Comparison of appositions on NPY cell types: ^**+**^*p*< .05; Comparison between genotypes: ^**§**^*p*< .05

### PV- and NPY-ir neuron numbers and densities in the BLA

**Fig. 8:**
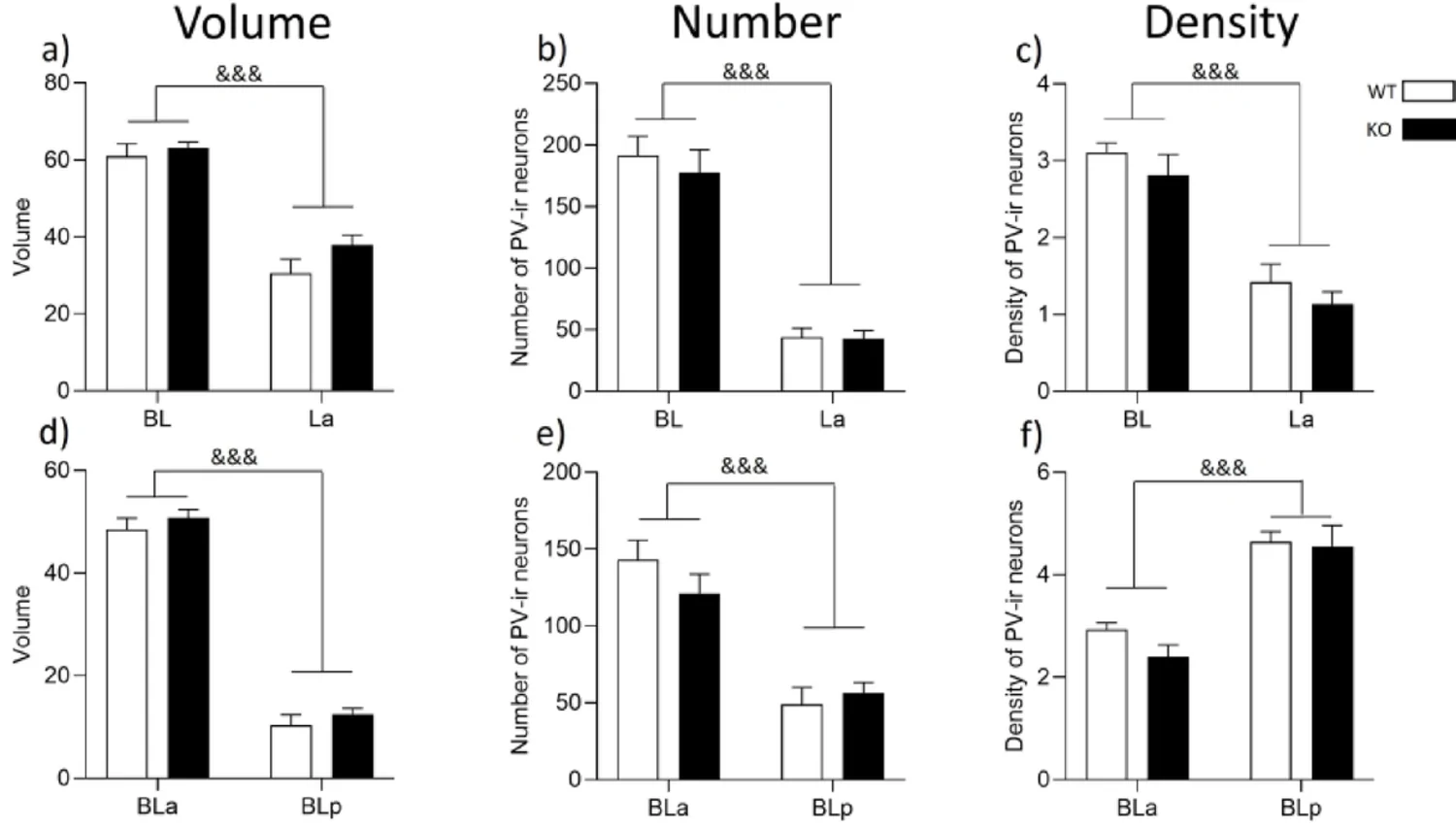
The numbers and densities of PV-ir neurons strongly depend on the amygdalar nuclei and subnuclei studied. Graphs illustrating the statistical results listed in Table 2.1 of Online Resource 2 regarding the analyzed volumes (x 10^6^ µm^3^) of the BL and La (**a**), the anterior and posterior part of the BL (BLa and BLp, respectively, **d**), as well as the numbers (**b**,** e**) and densities (**c**,** f**; neurons per 10^6^ µm^3^) of PV-ir cells in these amygdalar nuclei and subnuclei of 5-HTT wildtype (WT) and knockout (KO) mice. Data represent mean ± SEM. Comparison between different amygdala subregions: ^&&&^*p*< .001

**Fig. 9:**
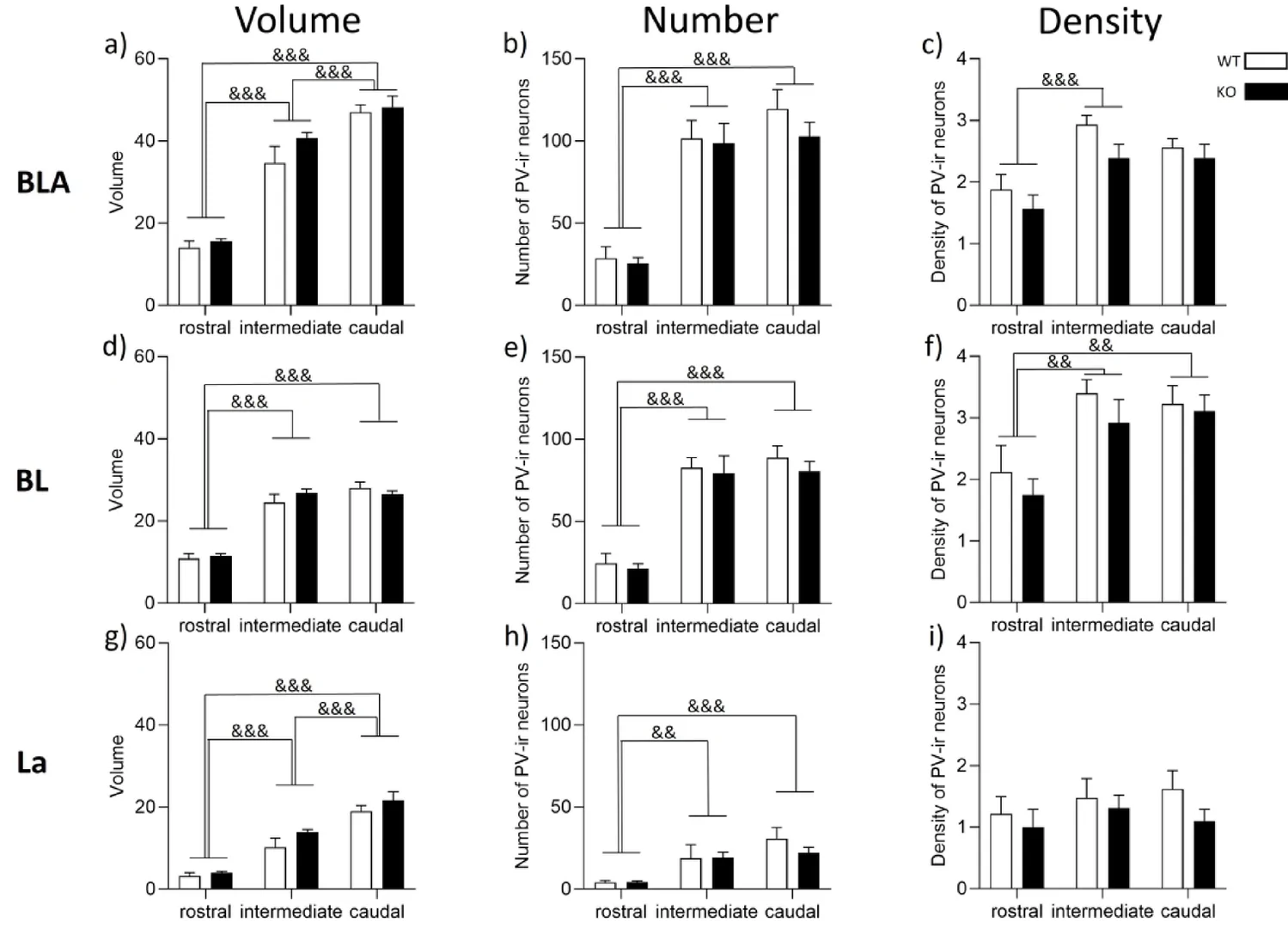
The numbers and densities of PV-ir neurons in the rostro-caudal axis of the BLA and its nuclei BL and La. Graphs illustrating the statistical results listed in Table 2.2 of Online Resource 2 regarding the analyzed volumes (x 10^6^ µm^3^) of the basolateral amygdala (BLA, **a**), the basolateral (BL, **d**) and lateral (La, **g**) nuclei of the amygdala as well as the numbers (**b**,** e**, **h**) and densities (**c**,** f**, **i**; neurons per 10^6^ µm^3^) of PV-ir neurons in these amygdalar nuclei in 5-HTT wild-type (WT) and knockout (KO) mice along the rostro-caudal axis (rostral, intermediate, caudal). Data represent mean ± SEM. Comparison between different amygdalar regions: ^&&^*p*< .01, ^&&&^*p*< .001

**Fig. 10:**
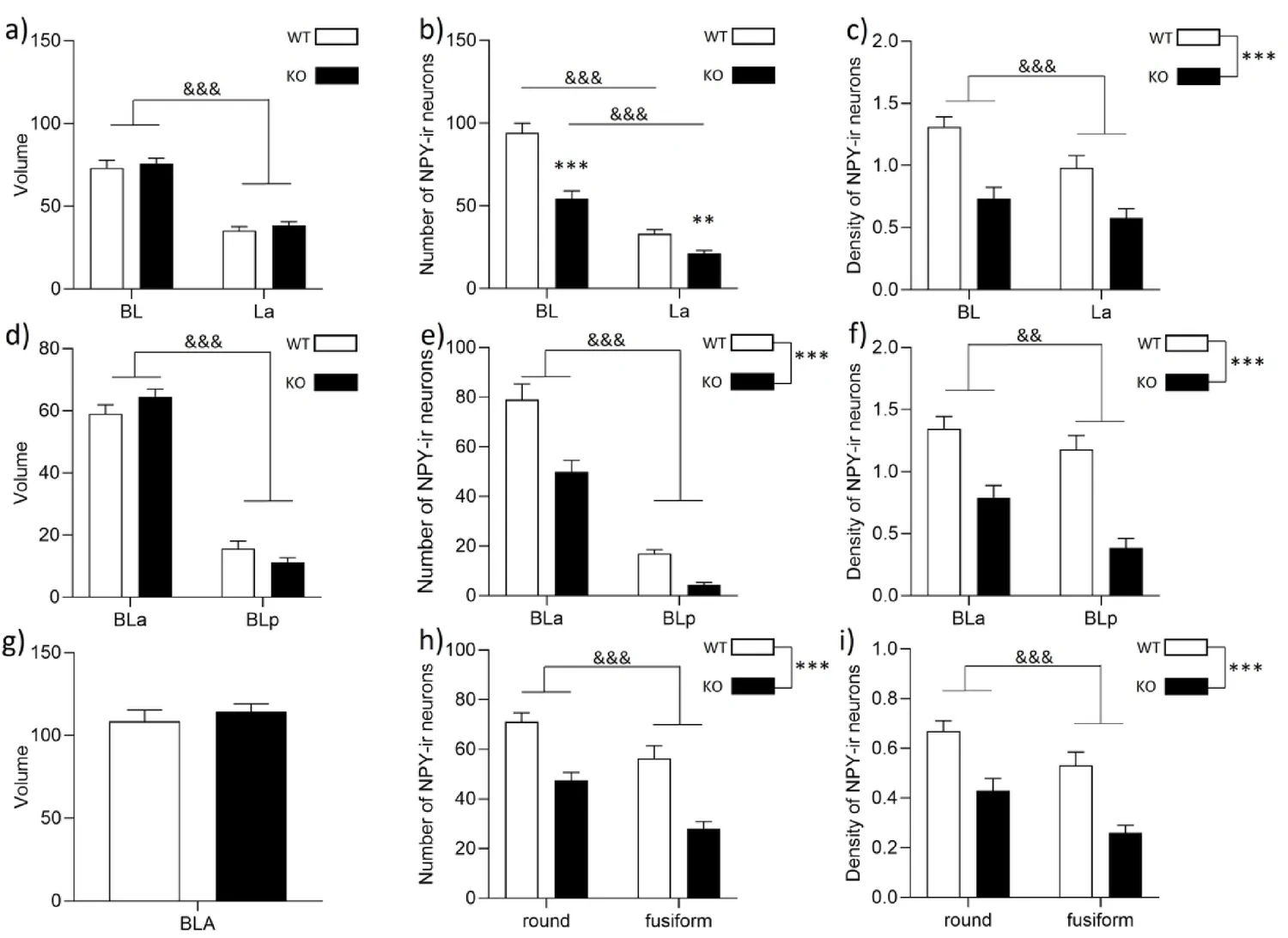
The *5-Htt* genotype has a strong influence on the numbers and densities of NPY-ir neurons. Graphs illustrating the statistical results described in Table 3.1 of Online Resource 3 regarding the analyzed volumes (x10^6^ µm^3^) of the basolateral amygdala (BLA, **g**), the basolateral (BL, **a**) and lateral nucleus (La, **a**), the anterior and posterior part of the BL (BLa and BLp, respectively, **d**), and the numbers (**b**,** e**) and densities (neurons per 10^6^ µm^3^; **c**,** f**) of NPY-ir cells in these amygdalar nuclei and subnuclei of 5-HTT wildtype (WT) and knockout (KO) mice. (**h**,** i**) These two graphs illustrate the numbers (h) and densities (i) of round and fusiform NPY-ir cells in the BLA as a function of the *5-Htt* genotype. Data represent mean ± SEM. Comparison between different amygdala nuclei or neuron types: ^&&^*p*< .01, ^&&&^*p*< .001. Comparison between the two *5-Htt* genotypes: ^**^p< .01, ^***^p< .001

**Fig. 11:**
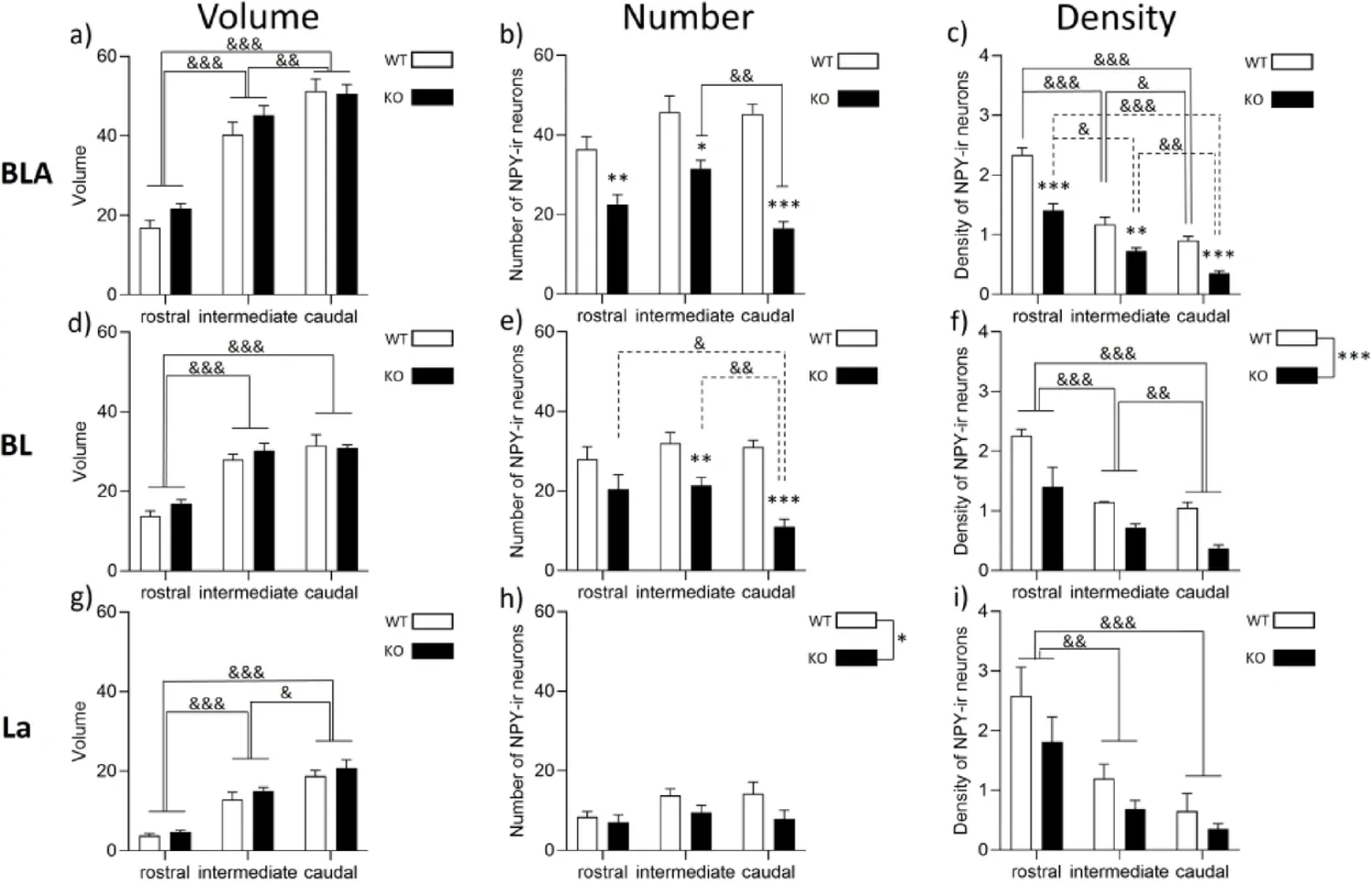
The numbers and densities of NPY-ir neurons in the rostro-caudal axis of the BLA and its nuclei BL and La. Graphs illustrating the statistical results described in Table 2.2 of Online Resource 3 regarding the analyzed volumes (x10^6^ µm^3^) of the basolateral amygdala (BLA, **a**), the basolateral (BL, **d**) and lateral (La, **g**) nuclei of the amygdala as well as the number (**b**, **e**, **h**) and density (**c**, **f**, **i**; neurons per 10^6^ µm^3^) of NPY-ir cells in these amygdalar nuclei of 5-HTT wild-type (WT) and knockout (KO) mice along the rostro-caudal axis (rostral, intermediate, caudal). Data represent mean ± SEM. Comparison between different amygdalar regions: ^&^*p*< .05, ^&&^*p*< .01, ^&&&^*p*< .001. Comparison between the two *5-Htt* genotypes: ^*^p< .05, ^**^p< .01, ^***^p< .001

Key results obtained from a mixed two-way analysis of variance (ANOVA) performed on quantitative data for the volumes analyzed for the BLA nuclei (BL and La) and BL subnuclei (BLa and BLp), the numbers of identified PV- and NPY-ir neurons and their densities in these nuclei/subnuclei of 5-HTT-WT and KO mice are summarized in tables of Online Resource 2 (PV; Table 2.1 and 2.2) and Online Resource 3 (NPY; Table 3.1 and 3.2), and illustrated in graphs (for PV: Figures 8 and 9 ; for NPY: Figures 10a-f and 11). In addition, numbers and densities of round and fusiform NPY-ir neurons were analyzed in the BLA of WT and 5-HTT KO mice (Online Resource 3: Table 3.1 and Fig. 10g-i).

*Analyzed volumes*. Comparison of the volumes of the different amygdalar nuclei analyzed for the numbers of PV- or NPY-ir neurons in six sections throughout the rostro-caudal extent for the entire BLA and in two sections each for the analyses of rostral (between − 0.80 and − 1.5 mm from bregma), intermediate (between − 1.5 and − 2.2 mm) and caudal (between − 2.2 and − 2.9 mm) regions of the BLA as well as of the two nuclei BL and La and the two subnuclei BLa and BLp revealed only significant main effects of the respective amygdalar nuclei/subnuclei or regions, no main effect of the *5-Htt* genotype and no interaction between nuclei or regions and *5-Htt* genotype (Online resource 2 and 3, Figs. 8, 9, 10 and 11). The volume of the BL analyzed for PV-ir or NPY-ir neurons in both genotypes is about twice as large as that of the La [PV: main effect of nuclei (F(1.15) = 390.701, *p* < .001); NPY: main effect of nuclei (F(1.15) = 427.698, *p* < .001)], and the volume analyzed for the anterior part of the BL (BLa) also differs in size from that analyzed for the posterior part of the BL (BLp) [PV: main effect of subnuclei (F(1.11) = 420.11, *p* < .001); NPY: main effect of subnuclei (F(1.14) = 788.589, *p* < .001)]. In both genotypes, the analyzed volumes of the rostral region of the BLA and of its nuclei BL and La are smaller than those of the intermediate and caudal region (PV: Online Resource 2 Table 2.2 and Fig. 9; NPY: Online Resource 3 Table 3.2 and Fig. 11). Although no statistical comparison was performed, these findings, along with additional data from Online Resources 2 and 3, appear to indicate that the volumes analyzed of the various amygdalar nuclei and regions in the sections labeled with PV antibodies and in the sections labeled with NPY antibodies yields comparable values for both genotypes.

*PV-ir neurons*. As shown in Online Resource 2 Table 2.1 and illustrated in Fig. 8a, b and c the number of PV-ir neurons is more than four times higher in the BL than in the La [F(1.15) = 259.583, *p* < .001], while the analyzed volume is only about twice the size [F(1.15) = 390.701, *p* < .001], without significant *5-Htt*-genotype differences [F(1.15) = 1.608, *p*= .225]. Accordingly, the BL exhibits a significantly higher density of PV-ir neurons compared to the La in both genotypes [F(1.15) = 193.978, *p* < .001].

Within the BL, PV-ir neurons are not homogeneously distributed: As shown in Online Resource 2 Table 2.1 and Fig. 8d, e, f, the number of PV-ir neurons in the BLa is significantly higher than in the BLp [F(F1.11) = 139.661, *p* < .001], but the BLa has a significantly lower density than the BLp [F(1.11) = 117.499, *p* < .001; Fig. 7b]. This is because the analyzed volume of the Bla is more than four times that of the BLp [F(F1.11) = 420.111, *p* < .001], while the number of PV-ir neurons is only about twice as high.

As depicted in Online resource 2 Table 2.2 and Fig. 9, the analyzed volumes of the entire BLA and of the BL and La along the rostral-caudal axis are significantly larger in the intermediate and caudal regions compared to the rostral region, and the volumes analyzed for the caudal region of the BLA and La are significantly larger than those of the intermediate region [BLA: Mixed ANOVA with F(2.12) = 201.722, *p* < .001, and subsequent Bonferroni multiple comparison tests revealed rostral vs. intermediate: *p* < .001; rostral vs. caudal: *p* < .001; intermediate vs. caudal: *p* < .001; BL: Mixed ANOVA with F(2.12) = 119.433, *p* < .001, and subsequent Bonferroni multiple comparison tests revealed rostral vs. intermediate: *p* < .001; rostral vs. caudal: *p* < .001; intermediate vs. caudal: *p* = .771; La: Mixed ANOVA with F(2.12) = 114.298, *p* < .001, and subsequent Bonferroni multiple comparison tests revealed rostral vs. intermediate: *p* < .001; rostral vs. caudal: *p* < .001; intermediate vs. caudal: *p* < .001]. In all cases, mixed ANOVA did not detect analyzed volume differences between mice of different *5-Htt* genotypes, and no gene by region interactions (Online resource 2 Table 2.2). As shown in Fig. 9b, e, h the number of PV-ir neurons is increased in the intermediate and also the caudal region compared to the rostral region, but is not increased in the caudal compared to the intermediate region of the BLA, BL and La [BLA: Mixed ANOVA with F(2.12) = 83.212, *p* < .001, and subsequent Bonferroni multiple comparison tests revealed rostral vs. intermediate: *p* < .001; rostral vs. caudal: *p* < .001; intermediate vs. caudal: *p* = .447; BL: Mixed ANOVA with F(2.12) = 94.576, *p* < .001, and subsequent Bonferroni multiple comparison tests revealed rostral vs. intermediate: *p* < .001; rostral vs. caudal: *p* < .001; intermediate vs. caudal: *p* = 1.0; La: Mixed ANOVA with F(2.12) = 12.812, *p* < .001, and subsequent Bonferroni multiple comparison tests revealed rostral vs. intermediate: *p* = .006; rostral vs. caudal: *p* < .001; intermediate vs. caudal: *p* = .602]. Mixed ANOVA did not detect differences in the analyzed parameters between the genotypes (Online resource 2).

With regard to the density of PV-ir neurons in the BLA, a significantly higher density is observed in the intermediate compared to the rostral region [Fig. 9c, Online resource 2 Table 2.2; Mixed ANOVA with F(2.12) = 10.821, *p* < .001, and subsequent Bonferroni multiple comparison tests revealed rostral vs. intermediate: *p* < .001; rostral vs. caudal: *p* = .057; intermediate vs. caudal: *p* = .490]. In the BL a similar pattern of PV-ir neuron density on different rostro-caudal axis regions was shown [F(2.12) = 14.877, *p* < .001, and subsequent Bonferroni multiple comparison tests revealed rostral vs. intermediate: *p* = .002; rostral vs. caudal: *p* = .004; intermediate vs. caudal: *p* = 1.0] (Fig. 9f, Online Resource 2 Table 2.2). Only in the La the Mixed ANOVA revealed no significant differences in the density of PV-ir neurons [F(2.23) = 0.698, *p* = .506] (Fig. 9i, Online Resource Table 2.2). Although mean values of PV-ir neuron densities throughout nuclei and subnuclei in 5-HTT KO are slightly lower than in WT mice, the differences do not reach significance (Online Resource 2 Table 2.2 and Fig. 11).

*NPY-ir neurons.* As shown in Table 3.1 of Online Resource 3 and illustrated in Fig. 10, the number of NPY-ir neurons is about three times higher in the BL than in the La [main effect of nuclei: F(1.15) = 175.455, *p* < .001], with a significant *5-Htt*-genotype difference [F(1.15) = 32.345, *p* < .001] and a significant gene by nuclei interaction [F(1.15) = 15.749, *p* = .001]. Bonferroni multiple comparison tests revealed that, in both WT and 5-HTT KO mice, there are significantly more NPY-ir neurons in the BL than in the La (*p* < .001) and that 5-HTT-KO mice have significantly fewer neurons in the BL and La compared to WT mice (BL WT vs. BL KO: *p* < .001; La WT vs. La KO: *p* = .001). The density of NPY-ir neurons is significantly higher in the BL than in the La, regardless of genotype [main effect of nuclei: F(1.15) = 21.499, *p* < .001] and 5-HTT-KO mice exhibit a significantly lower density compared to WT mice [main effect of genotype: F(1.15) = 18.370, *p* < .001], but no gene by nuclei interaction [F(1.15) = 2.956, *p* = .106].

As shown in Online Resource 3 Table 3.1 and Fig. 10, the analyzed volume of the Bla is significantly larger than the analyzed volume of the BLp [F(F1.14) = 788.589, *p* < .001]; the number of NPY-ir neurons in BLa is also significantly higher than in the BLp [F(F1.14) = 166.791, *p* < .001], and the BLa has a significantly higher density than BLp [F(1.11) = 15307, *p* = .002]. Additionally, statistical analysis revealed a highly significant *5-Htt*-genotype effect of the number of NPY-ir neurons [F(1.15) = 28.596, *p* < .001] and of the density of NPY-ir neurons [F(1.15) = 34.058, *p* < .001], in both cases without significant gene by subnuclei interactions [number: F(1.15) = 3.929, *p* = .067; density: F(1.15) = 2.560, *p* = .132].

With respect to the morphological subtypes of NPY-ir neurons, significantly more round than fusiform neurons are found in the BLA of both genotypes [F(1.15) = 32.480, *p* < .001] and there are significantly less NPY-neurons of both types found in 5-HTT KO mice [F(1.15) = 32.345, *p* < .001]. The density of round neurons is higher than that of fusiform neurons [F(1.15) = 27.858, *p* < .001] and there are significantly less NPY-ir neurons of both types found in 5-HTT KO mice [F(1.15) = 20.514, *p* < .001]. In these cases, no significant gene by nuclei interaction was detected (Online resource 3 Table 3.1 and Fig. 10h, i).

As shown in Fig. 11a, d, g (and described in Online Resource 3 Table 3.2), comparing different rostro-caudal levels of the amygdala the analyzed volumes of the BLA and its nuclei significantly increases from rostral to intermediate, from rostral to caudal, and in the case of BLA and La also from intermediate to caudal parts [BLA: Mixed ANOVA with F(2.13) = 142.186, *p* < .001, and subsequent Bonferroni multiple comparison tests revealed rostral vs. intermediate: *p* < .001; rostral vs. caudal: *p* < .001; intermediate vs. caudal: *p* = .009; BL: Mixed ANOVA with F(2.13) = 74.153, *p* < .001, and subsequent Bonferroni multiple comparison tests revealed rostral vs. intermediate: *p* < .001; rostral vs. caudal: *p* < .001; intermediate vs. caudal: *p* = .663; La: Mixed ANOVA with F(2.13) = 62.474, *p* < .001, and subsequent Bonferroni multiple comparison tests revealed rostral vs. intermediate: *p* < .001; rostral vs. caudal: *p* < .001; intermediate vs. caudal: *p* = .013]. In all cases, mixed ANOVA did not detect analyzed volume differences between different *5-Htt* genotypes, and no gene by region interactions.

As also shown in Table 3.2 of Online Resource 3, and illustrated in Fig. 11b, e, h, statistical analyses of the numbers of NPY-ir cells detected significant main effects of region (= different levels along the rostro-caudal axis) in the BLA [F(2.13) = 7.085, *p* = .003] and in the BL [F(2.13) = 4.519, *p* = .020], but not in the La [F(2.13) = 1.836, *p* = .178], but also main effects of genotype in the BLA [F(2.13) = 32.345, *p* < .001], in the BL [F(2.13) = 16.857, *p* = .001], and in the La [F(2.13) = 6.318, *p* = .025], as well as significant gene by region interactions in the BLA [F(2.13) = 5.166, *p* = .012], BL [F(2.13) = 5.797, *p* = .008], but not in the La [F(2.13)=0.684, *p* = .513]. In consequence, Bonferroni multiple comparison tests were performed and clearly demonstrate that in almost all parts of the rostro-caudal axis of all investigated amygdalar regions the numbers of NPY-ir neurons are lower in 5-HTT KO compared to WT mice and that regional differences are primarily detected in 5-HTT KO mice.

With regard to the densities of NPY-ir neurons in the BLA, BL as well as in the La, it is noted that they are highest in the rostral region and decrease in a caudal direction (Fig. 11c, f, i; Online resource 3 Table 3.2). Statistical analyses of the density of NPY-ir cells detected significant main effects of region in the BLA [F(2.13) = 113.917, *p* < .001], in the BL [F(2.13) = 48.616, *p* < .001], and in the La [F(2.13) = 17.631, *p* < .001], but also main effects of genotype in the BLA [F(2.13) = 48.618, *p* < .001], in the BL [F(2.13) = 21.089, *p* < .001], but not in the La [F(2.13) = 2.684, *p* = .124], as well as a significant gene by region interaction in the BLA [F(2.13) = 12.162, *p* < .001], but not in the BL [F(2.13) = 1.506, *p* = .243] and in the La [F(2.13)=0.317, *p* = .731]. Consequently, Bonferroni multiple comparison tests were performed and clearly demonstrate that in addition to the fact that highest levels of NPY-ir neuron density is detected in the rostral region of all investigated amygdalar nuclei with a decrease in a caudal direction, a significantly decreased density of NPY-ir neurons is noted in the BLA and BL. The differences detected in 5-HTT KO compared to WT mice for the different regions of the La do not reach significance (Table 3.2 of Online Resource 3, Fig. 11i), albeit a significantly lower NPY-ir neuron number and density is found in the 5-HTT KO mice for the entire La (Fig. 10b, c).

### Gene expression studies

**Fig. 12:**
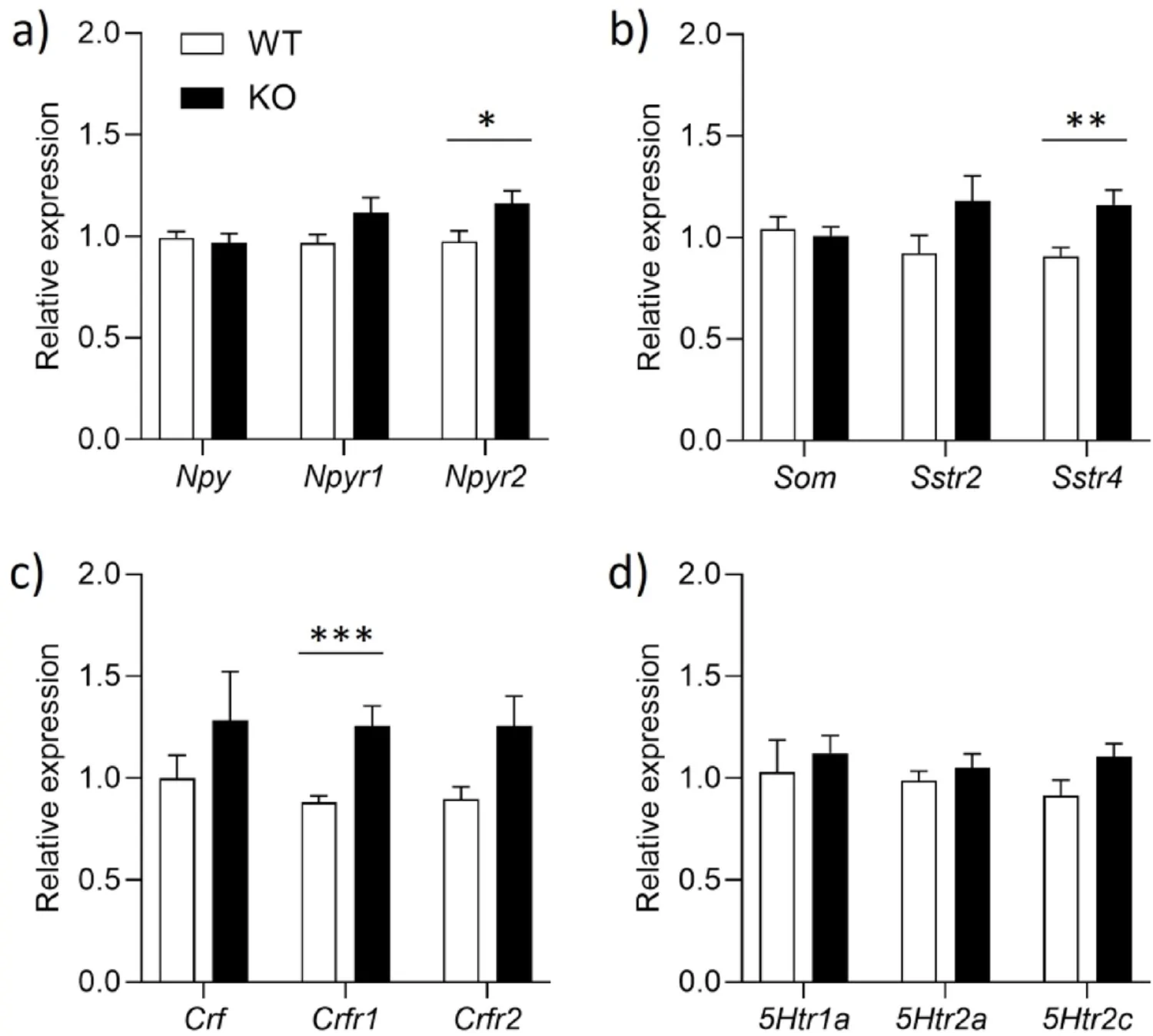
Expression levels of genes related to the NPY (**a**), SOM (**b**), CRF (**c**) and 5-HT systems (**d**) in the BLA of WT and 5-HTT KO mice. Expression was assessed by qPCR using microdissected BLA tissue. Significant increases in relative expression levels of *Npyr2*, *Sstr4*, and *Crfr1* are detected in 5-HTT KO compared to WT mice. Relative expression levels of the mRNA coding for the peptides NPY, SOM, and CRF did not show alterations. Relative mRNA expression levels of the serotonin receptors *5Htr1a*, -*2a* and *− 2c* were also unchanged

To assess possible differences in mRNA expression levels of * Npy*, *Som*, and of the anxiogenic peptide *Crf* and their receptors (*Npyr1* and *− 2*, *Sstr2* and *− 4*, *Crfr1* and *− 2*), qPCR analyses were carried out on laser-microdissected BLA tissue from mice of the different genotypes. Additionally, mRNA expression levels of 5-HT receptors *5Htr1a*, *-2a* and − *2c* were studied. Significant increases were found for *Npyr2*, *Sstr4* and *Crfr1*. The mRNA coding for the three peptides showed no differences. Changes were also not observed in the expression levels of the three investigated 5-HT receptors (Fig. 12; Online Resource 4).

## Discussion

The present study documents that life-long 5-HT imbalance in 5-HTT KO mice affects the NPY producing NPNs of the BLA nuclei: compared to WT mice, the numbers and densities of neurons which are identified as immunoreactive for the “anxiolytic” peptide NPY (Kask et al. 2002; Truitt et al. 2009; Yilmazer-Hanke et al. 2004) are significantly reduced in 5-HTT KO mice. In contrast, numbers and densities of PV-ir neurons in the BLA nuclei appear unaltered. Concerning interrelations between PV- and NPY-ir NPNs and catecholaminergic afferents, TH-ir, presumably dopaminergic perisomatic appositions are similar in WT and 5-HTT KO mice. Furthermore, on the gene expression level, significantly increased mRNA levels are observed in 5-HTT KO mice coding for specific peptide receptors presumably involved in BLA processing of stress- and anxiety-inducing stimuli (NPYR2, SSTR4 and CRFR1; Giesbrecht et al. 2010; Scheich et al. 2016). These findings indicate that dysregulation of the BLA NPY-system may contribute to the increased stress- and anxiety related behavior of 5-HTT KO mice (Murphy and Lesch 2008), and add to the growing body of evidence implicating inhibitory networks of the BLA, particularly those involving the NPY-producing NPNs of the BL and La, as important players in the modulation of emotional stimulus processing and anxiety (Yilmazer-Hanke et al. 2002; Truitt et al. 2009; Prager et al. 2016).

### Morphology of PV- and NPY-ir BLA neurons, colocalization of NPY with PV or SOM

The morphological characteristics of the three types of PV-ir BLA neurons documented in WT and 5-HTT KO mice in the present study are similar to what has been previously described in rats (Kemppainen and Pitkänen 2000) and monkeys (Pitkänen and Amaral, 1993). In morphological and combined electrophysiological/morphological investigations, PV-ir INs in the BLA of rodents were identified as basket or axoaxonic (chandelier) neurons which displayed a multipolar somatic morphology (McDonald and Betette 2001; Vereczki et al. 2021); an analysis specifically aimed at identifying the functional properties of the three morphological subtypes of BLA PV-INs has not been carried out yet. There was no colocalization observed of PV- and NPY-IR in our material. This is in accordance with findings in rats (McDonald 2023). However, in an elegant recent study using a combination of transgenic strategies and viral vectors to label, among other inhibitory neurons, NPY-producing neurons in the mouse BLA, Vereczki et al. (2021) reported that ~ 25% of BLA-NPNs expressing an NPY-reporter protein coexpress PV. On the basis of morphological features and electrophysiological properties of these neurons, the authors suggested that PV/NPY-producing INs, like other PV-producing INs, are PN-targeting basket or axoaxonic cells. A possible reason for the fact that we did not observe PV/NPY-labeled neurons in our material could be that production of NPY peptide in these neurons is very low and cannot be detected in neuronal somata by the immunolabeling methods applied in the present study: The immunoenzyme and immunofluorescence labeling approaches to assess the NPY content of NPNs are presumably less sensitive than detection of reporter proteins for the different peptides or proteins produced in various neuronal populations in transgenic animals and/or by transfection using viral vectors (Vereczki et al. 2021). On the other hand, immunodetection of the peptides circumvents questions of expression specificity and enables detection of the NPN-content of the peptide or protein in question produced in WT mice and 5-HTT KO mice without genetic manipulations. In any case, neurons identified as NPY-ir in the present study in both genotypes may represent neurons with a rather high content of the peptide. The characteristic labeling of “cytoplasmic streets” or “spots” in NPY-ir neurons most likely represents nascent NPY-peptide in Golgi stacks (Takagi et al. 1983; Asan et al. 1998).

The SOM/NPY dual labelings enabled us to tentatively identify morphological subtypes of NPY-ir neurons in WT and 5-HTT KO mice. Most double-labeled neurons possess elongated fusiform, sometimes oval or polygonal somata, indicating that morphologically similar neurons in the immunoenzyme labelings (“fusiform neurons”) represent dendrite-targeting SOM/NPY-INs or SOM/NPY-projection neurons, which have both been reported to possess elongated or fusiform somata (Bocchio et al. 2016b; Vereczki et al. 2021). Again, the labeling of cytoplasmic compartments most likely represents Golgi-stacks with a rather high content of nascent peptides (Takagi et al. 1983; Asan et al. 1998). Single NPY-labeled neuronal somata were mostly small and round, and thus morphologically resemble NPY-ir NGFCs (Manko et al. 2012; Overstreet-Wadiche and McBain 2015; Ozsvar et al. 2024). These neurons possess spherical cell bodies and have been reported to usually lack SOM-IR (Perrenoud et al. 2012; Vereczki et al. 2021). Vereczki et al. (2021) found that almost half (ca. 45%) of NPY reporter expressing neurons were NGFCs in the BLA. Thus, although it has to be cautioned that specific identification of NGFCs was not carried out in our investigation, it appears reasonable to assume that many of the NPY-ir cells with round somata in our immunoenzyme-labeled material of WT and 5-HTT KO mice are NGFCs. Unfortunately, it was not possible to perform quantitative analyses of the SOM/NPY dual labelled sections and thus to assess whether the ratios of the different NPY-ir neuron types are altered in 5-HTT KO compared to WT mice. Future investigations using more sensitive methods to neurochemically characterize the different neuron types, for instance detection of peptide mRNA or crossing of mice expressing transgenic reporters for the peptides with 5-HTT KO mice, will have to address this aspect.

### Interrelations of PV- and NPY-ir BLA neurons with monoaminergic afferents

In the rat, PV-ir neurons have been shown to receive numerous perisomatic synapses, many of which are symmetric (Müller et al. 2005). Electron microscopy showed that narrow varicosities of serotonergic and dopaminergic afferents form multiple appositions on PV-ir perikarya, and small synaptic contacts were identified in most of them (Muller et al. 2007b; Pinard et al. 2008). NPY-ir BL neurons in the rat are also contacted by serotonergic and TH-ir afferents at their perikarya, and small synaptic contacts have been detected (Asan et al. 1998; Bonn et al. 2013). Our findings document perisomatic appositions of both monoaminergic afferent types on all morphological subgroups of PV-ir and NPY-ir BLA neurons in the WT and also in 5-HTT KO mice. Monoamines are known to mediate their effects not only via synaptic, but also via volume transmission (Bunin and Wightman 1998; Zoli et al. 1999; Fuxe et al. 2007; Gianni et al.^2023^; Özcete et al. 2024). Although monoaminergic afferents also form contacts with dendrites and axon terminals (Asan 1998; Müller et al. 2007b; Bonn et al. 2013; McDonald 2023), their perisomatic appositions are in a particularly powerful position to influence the activity of their target neurons either directly or via binding to presynaptic receptors on terminals of other (mostly GABAergic) neurons forming contacts on the same somata.

Our quantitative data indicate that apposition frequencies of serotonergic afferents on PV- and NPY-ir somata are correlated with the 5-HTT-ir fiber densities observed in the WT BL and La, respectively, Moreover, both perisomatic apposition frequencies, and the reduction in apposition frequencies in the La compared to the BL, are similar for PV- and NPY-ir neurons in the WT. Perisomatic appositions formed by 5-HT-ir fibers were observed on both types of BLA-NPNs also in the 5-HTT KO mice. In the rat BNC, 5-HT1A and 5-HT2A receptors have been found in PV-ir neurons (McDonald and Mascagni 2007; Bombardi 2014; McDonald 2023). Expression of *5-Ht1a* and *5-Ht2c* receptors has been observed on mostly separate subpopulations of NPY-ir neurons (Bonn et al. 2013). While 5-HT1A is an inhibitory receptor, 5-HT2 receptors mediate neuronal excitation (Asan et al. 2013). Provided that receptor expression in mice is similar, these results suggest that one subpopulation of NPY-NPNs can be activated by serotonergic innervation, another can be inhibited. Whether NPY-ir NPN subpopulations expressing different 5-HT receptors correspond to the described subpopulations of NPY-NPNs (dendrite-targeting INs, projection neurons, NGFCs) and whether there are differences in receptor expression between NPY-NPNs in WT and 5-HTT KO mice remains to be determined.

PV- and NPY-ir neurons in WT mice are contacted by TH-ir, presumably predominantly dopaminergic afferents in the BLA. Previous ultrastructural studies have documented that PV-ir neurons, but not calretinin-immunoreactive neurons, were prominent targets of dopaminergic terminals in the basolateral complex of the rat (Pinard et al. 2008). Bissiere et al. (2003) reported that dopaminergic innervation can modulate feedforward inhibition in the La. A significant proportion of PV neurons in the BL and La in the rat have been shown to express the Dopamin D2 receptor (Rowniak et al. 2017). Dopamine receptors in NPY-ir BLA neurons have not been identified yet (McDonald 2023). The findings described for the innervation of PV-ir and NPY-ir neurons by TH-ir fibers in the WT mouse BLA also apply to the 5-HTT KO mice, with minor genotype differences in the innervation of NPY-ir round neurons (Table 1). This indicates that serotonin imbalance in development does not have a major impact on morphological parameters of the dopaminergic innervation of the BLA in 5-HTT KO mice.

### Quantitative analyses of PV- and NPY-ir neurons in the BLA in WT and 5-HTT KO mice

PV-ir neuron density in the entire BLA increase in a rostrocaudal direction with significantly higher densities of neurons in the intermediate (-1.5 to -2.2 mm from bregma) compared to the rostral level. This agrees with the observation of Vereczki et al. (2021), who reported that the proportion of inhibitory (GABAergic) cells among all neurons of the BLA peaked at -2.1 mm from bregma. Significant differences are observed between BL and La as well as between Bla and BLp in both genotypes, but are absent between the genotypes in the respective nuclei/subnuclei, indicating that 5-HTT deficiency does not influence these parameters.

NPY-ir neuron density in WT mice is higher in the BL than in the La and decreases in a rostro-caudal direction. A higher percentage of NPY-neurons was found in the BL than in the La in mice also by Vereczki et al. (2021). In contrast to PV-ir neurons, significantly less neurons identified as NPY-ir are observed in the 5-HTT KO animals throughout the La and BL, indicating that life-long 5-HT imbalance significantly affects the NPY-producing population of BLA-NPNs.

### Expression of anxiety-related peptides and receptors

Our gene expression studies showed that there is neither a change for *Npy* nor for *Som* or *Crf* mRNA in the BLA of 5-HTT KO animals. This argues against the proposition that the reduction in the number of BLA NPNs with detectable NPY-IR in the 5-HTT KO mice is due to the absence of NPNs capable of producing NPY, caused for instance by a dysfunctional migration of NPY-NPN precursors from their subpallial areas of origin into the BLA (Real et al. 2009; Overstreet-Wadiche and McBain, 2015) under conditions of life-long 5-HT imbalance. It appears rather that, in 5-HTT-deficient animals, the translation of the peptide is reduced in BLA-NPNs which transcribe its mRNA and are, in principle, capable of producing NPY, which they may do at a higher rate in the WT than the 5-HTT KO mice. The observation that in our material NPY-IR is often concentrated in somatic compartments presumably representing Golgi-stacks (Takagi et al. 1983; Asan 1998) suggests that the respective neurons are engaged in production of the peptide. It remains to be analyzed whether BLA-NPNs can increase their NPY production on demand or whether further NPNs can be recruited to produce NPY, perhaps in stressful situations, and, if so, how this demand is relayed to the neurons. Interestingly, a recent study documented a reduction of NPY-ir NPN numbers in the BLA of aged rats. This could be reversed by intracerebroventricular injection of NGF, which also led to an increased density of cholinergic axonal varicosities in the BLA (Pereira et al. 2024). Another recent study on the NPY-IN system in the ventral hippocampus showed that extinction training increased the fraction of NPY-transcript positive cells (Wu et al. 2026). NPY production in cortical and hippocampal INs has been shown to be influenced by extrinsic innervation from cholinergic systems (Milner et al. 1997 ; Zhang et al. 1998), and an activity-dependent increase of NPY levels in hippocampal interneurons has been shown after experimentally induced seizures (Sperk 2026). Monoaminergic afferents, which influence neuronal activity via G-protein-coupled receptors, may thus also be involved in regulating peptide production. In any case, increased mRNA levels of *Npyr2* support the observation that the BLA-NPY-system is indeed affected by life-long 5-HT imbalance. Increased mRNA of *Crfr1*, a receptor that mediates anxiogenic effects (Giesbrecht et al. 2010), and of *Sstr4*, which presumably mediates anxiolysis (Scheich et al. 2016) indicates that other peptidergic systems may also be involved in altered amygdala processing in 5-HTT KO mice. The finding that mRNA of the 5-HT receptors analyzed in the present study did not display differences in 5-HTT KO compared to WT mice does not argue against a possible dysfunctionality of receptor-mediated signal transduction, since transduction efficiency of receptors not only depends on the amount of receptor produced, but also on molecular interactions with various other membrane or cytoplasmic molecules (Bohn and Schmid 2010, Chruscicka et al. 2019; Xu et al. 2021). This, of course, also applies to the other receptors analyzed in the present study.

### Functional implications

Neuroplasticity allows adaptation to changed environmental conditions. The BLA, particularly the La, is considered to be the main input station of the amygdala. Various informations from cortical and subcortical structures are integrated here. Thus, it is not surprising that the BLA is a place of intense neuroplasticity. Most classic studies of conditioned fear focus on NMDA- and AMPA-receptor-dependent long-term potentiation (LTP) of glutamatergic PNs, but in recent years it has become increasingly obvious that GABAergic NPNs play a decisive role in plasticity processes as well (Ehrlich et al. 2009; Prager et al. 2016; Fu et al. 2024).

Analyses of the synaptology have documented that networks formed by different populations of GABAergic NPNs fulfill distinct tasks in information processing in BL and La via the regulation of PN activity (Hajos 2021), and thus presumably of PN plasticity processes. Plasticity is also exhibited by inhibitory INs: Recent studies have documented that, for instance, PV-, SOM- and VIP-producing BLA IN-types exhibit unique activity patterns in conditioned fear and extinction learning, and that PV- and SOM-INs dynamically track behavioral-state transitions across learning, with SOM-INs particularly facilitating safety-related processing (Stujenske et al. 2022; Baldi et al. 2025; Favila et al. 2025).

The synaptic contacts of SOM-ir INs, and thus most likely of NPY-ir INs coexpressing SOM, are located at distal dendrites of pyramidal cells (Krabbe et al. 2018; Hajos 2021). These IN inputs are ideally positioned to modulate excitatory synaptic transmission also directed mainly at distal dendrites and spines of PNs, and thus may influence PN dendritic and spine plasticity (Krabbe et al. 2018). NPY-ir NGFCs are considered one of the major contributors to slow-phasic and tonic GABA release in the BLA, resulting in both GABA-A and GABA-B receptor mediated inhibition of PNs at their dendritic compartments (Hajos 2021; Ozsvar et al. 2024). However, their exact contribution to BLA circuit operation remains unknown, as are possible effects of NPY released from these neurons (Ozsvar et al. 2024). Investigations of the activity and/or of the NPY-production in BLA-INs of 5-HTT KO mice under different conditions may yield insights into these questions.

In experimentally naïve 5-HTT KO mice, number and density of spines on distal dendrites of La and BL PNs are significantly higher than in WT animals (Wellman et al. 2007; Nietzer et al. 2011). This has been suggested to be a morphological correlate for an increased excitability of the BLA-PNs and, as a consequence, the per se anxious phenotype of these animals (as tested, for instance, in the dark-light test, Jansen et al. 2010). Based on the findings of the present study, it could be suggested that a comparatively low level of NPY transmission from SOM/NPY-producing INs and/or from NGFCs in experimentally naïve 5-HTT KO mice may lead to reduced dendritic inhibition and thus an increased excitatory drive on PN dendrites, which may result in higher spine numbers. In addition, Nietzer et al. (2011) showed that, in WT mice, social defeat stress, induced by repeated loser experience in the social defeat paradigm, leads to a significant increase in spine density to the level observed in naïve 5-HTT KO mice, whereas no further increase was observed in stressed compared to non-stressed 5-HTT KO mice, indicating a lack of dendritic plasticity. In this respect, following suggestions by Mackay et al. 2019, it could be suggested that in the WT mice NPY, released from GABAergic NPY-IN axons onto PN distal dendrites and, acting on presynaptic inhibitory NPYR2 autoreceptors, may induce selective disinhibition of dendritic domains under stress, enabling situation-adequate dendritic plasticity, perhaps as a “memory trace” of the stressful event. The relative scarcity of NPY in 5-HTT mice could reduce such plasticity. At the same time, non-social fear-related behavior (as measured by the elevated plus maze and the open field test) was significantly increased in 5-HTT KO mice previously subjected to social defeat stress while it was not increased in their WT counterparts (Jansen et al. 2010). This shows that socially stressed WT mice can still regulate behavior in non-social fear-related paradigms, possibly via inhibitory modulation of BLA PN excitation. In 5-HTT KO mice a dysfunctional inhibitory modulation of PN activity, perhaps additionally compromised due to stress-induced alterations for instance in monoaminergic transmission in the BLA, may result in an inability to adequately modulate excitation of BLA PNs leading to increased non-social fear-related behavior. The hypotheses generated from the findings of the present work will have to be tested in future investigations.

Of note, the absence of alterations in PV-ir neuron numbers in 5-HTT KO mice does not exclude effects of alterations in 5-HTT-expression on PV-ir neuron functions. Indeed, overexpression of 5-HTT in mice has been documented to result in reduced recruitment of PV-ir neurons in BL and La and in impaired fear-related behavior (Bocchio et al. 2015). As discussed above, PV neurons express the 5-HT2A receptor, and electrophysiological studies indicated that 5-HT-induced activation of PV-ir neurons and thus basic inhibition of principal cells was impaired due to reduction in 5-HT2A receptor function in 5-HTT overexpressing mice (Bocchio et al. 2015).

## Conclusion

Our findings show that the disrupted 5-HT homeostasis in 5-HTT KO mice leads to alterations of inhibitory networks involving NPY-NPNs in the BLA. Disturbances in the complex interplay of inhibition and disinhibition/excitation may be the result. Alterations in the network formed by NPY-ir neurons are clearly of particular physiological and clinical relevance. Similar changes in individuals who are carriers of the *S*-allele of the *5-HTT* gene with a resulting lower functional expression of 5-HTT could be contributing to the increased activity and responsiveness of the amygdala and, ultimately, to the altered processing of and response to emotional stimuli in these individuals.

## Supplementary Information

Below is the link to the electronic supplementary material.

Supplementary Material 1

## Data Availability

All data supporting the findings of this study are available within the paper and its Supplementary Information.

## References

[CR1] Almeida-Suhett CP, Prager EM, Pidoplichko V, Figueiredo TH, Marini AM, Li Z, Eiden LE, Braga MF (2014) Reduced GABAergic inhibition in the basolateral amygdala and the development of anxiety-like behaviors after mild traumatic brain injury. PLoS ONE 9(7):e102627. 10.1371/journal.pone.0102627 PMC4105413 25047645

[CR2] Asan E (1993) Comparative single and double immunolabelling with antisera against catecholamine biosynthetic enzymes: criteria for the identification of dopaminergic, noradrenergic and adrenergic structures in selected rat brain areas. Histochemistry 99(6):427–442. 10.1007/BF00274095 8104918

[CR3] Asan E (1997) Ultrastructural features of tyrosine-hydroxylase-immunoreactive afferents and their targets in the rat amygdala. Cell Tissue Res 288(3):449–469. 10.1007/s004410050832 9134859

[CR4] Asan E (1998) The catecholaminergic innervation of the rat amygdala. Adv Anat Embryol Cell Biol 142:1–118. 10.1007/978-3-642-72085-7 9586282

[CR5] Asan E, Steinke M, Lesch KP (2013) Serotonergic innervation of the amygdala: targets, receptors, and implications for stress and anxiety. Histochem Cell Biol 139(6):785–813. 10.1007/s00418-013-1081-1 23494464

[CR6] Baldi R, Muthuswamy S, Yasmin F, Quadir S, Zaidi D, Kwon M, Kandil I, Johnson K, Loomba N, Patel S (2025) Synaptic organization and associative learning-related activity of basolateral amygdala interneurons. Cell Rep 44(10):116295. 10.1016/j.celrep.2025.116295 PMC12588113 40971296

[CR7] Barkus C, Line SJ, Huber A, Capitao L, Lima J, Jennings K, Lowry J, Sharp T, Bannerman DM, McHugh SB (2014) Variation in serotonin transporter expression modulates fear-evoked hemodynamic responses and theta-frequency neuronal oscillations in the amygdala. Biol Psychiatry 75(11):901–908. 10.1016/j.biopsych.2013.09.003 PMC4032572 24120093

[CR8] Bengel D, Murphy DL, Andrews AM, Wichems CH, Feltner D, Heils A, Mossner R, Westphal H, Lesch KP (1998) Altered brain serotonin homeostasis and locomotor insensitivity to 3, 4-methylenedioxymethamphetamine (Ecstasy) in serotonin transporter-deficient mice. Mol Pharmacol 53(4):649–655. 10.1124/mol.53.4.649 9547354

[CR9] Bienvenu TC, Busti D, Magill PJ, Ferraguti F, Capogna M (2012) Cell-type-specific recruitment of amygdala interneurons to hippocampal theta rhythm and noxious stimuli in vivo. Neuron 74(6):1059–1074. 10.1016/j.neuron.2012.04.022 PMC3391683 22726836

[CR10] Bissiere S, Humeau Y, Luthi A (2003) Dopamine gates LTP induction in lateral amygdala by suppressing feedforward inhibition. Nat Neurosci 6(6):587–592. 10.1038/nn1058 12740581

[CR11] Bocchio M, Fucsina G, Oikonomidis L, McHugh SB, Bannerman DM, Sharp T, Capogna M (2015) Increased Serotonin Transporter Expression Reduces Fear and Recruitment of Parvalbumin Interneurons of the Amygdala. Neuropsychopharmacology 40(13):3015–3026. 10.1038/npp.2015.157 PMC4864439 26052039

[CR13] Bocchio M, Fisher SP, Unal G, Ellender TJ, Vyazovskiy VV, Capogna M (2016b) Sleep and Serotonin Modulate Paracapsular Nitric Oxide Synthase Expressing Neurons of the Amygdala. eNeuro 3(5). 10.1523/ENEURO.0177-16.2016 PMC5088777 27822504

[CR12] Bocchio M, McHugh SB, Bannerman DM, Sharp T, Capogna M (2016a) Serotonin, Amygdala and Fear: Assembling the Puzzle. Front Neural Circuits 10:24. 10.3389/fncir.2016.00024 PMC4820447 27092057

[CR14] Bohn LM, Schmid CL (2010) Serotonin receptor signaling and regulation via beta-arrestins. Crit Rev Biochem Mol Biol 45(6):555–566. 10.3109/10409238.2010.516741 PMC4776633 20925600

[CR15] Bombardi C (2014) Neuronal localization of the 5-HT2 receptor family in the amygdaloid complex. Front Pharmacol 5:68. 10.3389/fphar.2014.00068 PMC3988395 24782772

[CR16] Bompolaki M, Vantrease JE, DeJoseph MR, Tapia M, Colmers AP, W. F., Urban JH (2024) Activation of NPY Receptors in the BLA Inhibits Projections to the Bed Nucleus of the Stria Terminalis and Buffers Stress-Induced Decreases in Social Interaction in Male Rats. J Neurosci 44(34). 10.1523/JNEUROSCI.0228-24.2024 PMC11340280 39025677

[CR17] Bonn M, Schmitt A, Lesch KP, Van Bockstaele EJ, Asan E (2013) Serotonergic innervation and serotonin receptor expression of NPY-producing neurons in the rat lateral and basolateral amygdaloid nuclei. Brain Struct Funct 218(2):421–435. 10.1007/s00429-012-0406-5 PMC3580143 22527118

[CR18] Brooks SJ, Stein DJ (2015) A systematic review of the neural bases of psychotherapy for anxiety and related disorders. Dialogues Clin Neurosci 17(3):261–279. 10.31887/DCNS.2015.17.3/sbrooks PMC4610611 26487807

[CR19] Brunetti M, Sepede G, Mingoia G, Catani C, Ferretti A, Merla A, Del Gratta C, Romani GL, Babiloni C (2010) Elevated response of human amygdala to neutral stimuli in mild posttraumatic stress disorder: neural correlates of generalized emotional response. Neuroscience 168(3):670–679. 10.1016/j.neuroscience.2010.04.024 20416363

[CR20] Bunin MA, Wightman RM (1998) Quantitative evaluation of 5-hydroxytryptamine (serotonin) neuronal release and uptake: an investigation of extrasynaptic transmission. J Neurosci 18(13):4854–4860. 10.1523/JNEUROSCI.18-13-04854.1998 PMC6792557 9634551

[CR21] Chruscicka B, Fitzsimons W, Borroto-Escuela SE, Druelle DO, Stamou C, Nally P, Dinan K, Cryan TG, Fuxe JF, K., Schellekens H (2019) Attenuation of Oxytocin and Serotonin 2A Receptor Signaling through Novel Heteroreceptor Formation. ACS Chem Neurosci 10(7):3225–3240. 10.1021/acschemneuro.8b00665 31038917

[CR22] Cohen H, Liu T, Kozlovsky N, Kaplan Z, Zohar J, Mathe AA (2012) The neuropeptide Y (NPY)-ergic system is associated with behavioral resilience to stress exposure in an animal model of post-traumatic stress disorder. Neuropsychopharmacology 37(2):350–363. 10.1038/npp.2011.230 PMC3242318 21976046

[CR23] Colmers PLW, Antonoudiou P, Basu T, Coleman EM, He Y, Scapa G, Fuller P, Maguire J (2025) Loss of PV Interneurons in the BLA May Contribute to Altered Network and Behavioral States in Chronically Epileptic Mice. eNeuro 12(1). 10.1523/ENEURO.0482-23.2024 PMC11773627 39746805

[CR24] Courtiol E, Menezes EC, Teixeira CM (2021) Serotonergic regulation of the dopaminergic system: Implications for reward-related functions. Neurosci Biobehav Rev 128:282–293. 10.1016/j.neubiorev.2021.06.022 PMC8335358 34139249

[CR25] Cunha C, Smiley JF, Chuhma N, Shah R, Bleiwas C, Menezes EC, Seal RP, Edwards RH, Rayport S, Ansorge MS, Castellanos FX, Teixeira CM (2021) Perinatal interference with the serotonergic system affects VTA function in the adult via glutamate co-transmission. Mol Psychiatry 26(9):4795–4812. 10.1038/s41380-020-0763-z PMC7657958 32398719

[CR26] Davis P, Zaki Y, Maguire J, Reijmers LG (2017) Cellular and oscillatory substrates of fear extinction learning. Nat Neurosci 20(11):1624–1633. 10.1038/nn.4651 PMC5940487 28967909

[CR27] Drevets WC (2003) Neuroimaging abnormalities in the amygdala in mood disorders. Ann N Y Acad Sci 985:420–444. 10.1111/j.1749-6632.2003.tb07098.x 12724175

[CR28] Ehrlich I, Humeau Y, Grenier F, Ciocchi S, Herry C, Luthi A (2009) Amygdala inhibitory circuits and the control of fear memory. Neuron 62(6):757–771. 10.1016/j.neuron.2009.05.026 19555645

[CR29] Eliava M, Yilmazer-Hanke D, Asan E (2003) Interrelations between monoaminergic afferents and corticotropin-releasing factor-immunoreactive neurons in the rat central amygdaloid nucleus: ultrastructural evidence for dopaminergic control of amygdaloid stress systems. Histochem Cell Biol 120(3):183–197. 10.1007/s00418-003-0557-9 12910346

[CR30] Favila N, Marsico C, Pacheco J, Kenet CM, Escribano S, Bitterman B, Grundemann Y, Luthi J, A., Krabbe S (2025) Heterogeneous plasticity of amygdala interneurons in associative learning and extinction. Nat Commun 16(1):9926. 10.1038/s41467-025-66122-y PMC12614800 41219181

[CR31] Franklin KBJ, Paxinos G (1997) The Mouse Brain in Stereotaxic Coordinates. Academic

[CR32] Fu X, Tasker JG (2024) Neuromodulation of inhibitory synaptic transmission in the basolateral amygdala during fear and anxiety. Front Cell Neurosci 18:1421617. 10.3389/fncel.2024.1421617 PMC11236696 38994327

[CR33] Fuxe K, Dahlstrom A, Hoistad M, Marcellino D, Jansson A, Rivera A, Diaz-Cabiale Z, Jacobsen K, Tinner-Staines B, Hagman B, Leo G, Staines W, Guidolin D, Kehr J, Genedani S, Belluardo N, Agnati LF (2007) From the Golgi-Cajal mapping to the transmitter-based characterization of the neuronal networks leading to two modes of brain communication: wiring and volume transmission. Brain Res Rev 55(1):17–54. 10.1016/j.brainresrev.2007.02.009 17433836

[CR35] Garcia-Calero E, Martinez-de-la-Torre M, Puelles L (2020) A radial histogenetic model of the mouse pallial amygdala. Brain Struct Funct 225(7):1921–1956. 10.1007/s00429-020-02097-4 PMC7473974 32583144

[CR34] Garcia-Lopez M, Abellan A, Legaz I, Rubenstein JL, Puelles L, Medina L (2008) Histogenetic compartments of the mouse centromedial and extended amygdala based on gene expression patterns during development. J Comp Neurol 506(1):46–74. 10.1002/cne.21524 PMC4916653 17990271

[CR36] Gianni G, Pasqualetti M (2023) Wiring and Volume Transmission: An Overview of the Dual Modality for Serotonin Neurotransmission. ACS Chem Neurosci 14(23):4093–4104. 10.1021/acschemneuro.3c00648 PMC10704479 37966717

[CR37] Giesbrecht CJ, Mackay JP, Silveira HB, Urban JH, Colmers WF (2010) Countervailing modulation of Ih by neuropeptide Y and corticotrophin-releasing factor in basolateral amygdala as a possible mechanism for their effects on stress-related behaviors. J Neurosci 30(50):16970–16982. 10.1523/JNEUROSCI.2306-10.2010 PMC3432911 21159967

[CR38] Godlewska BR, Browning M, Norbury R, Cowen PJ, Harmer CJ (2016) Early changes in emotional processing as a marker of clinical response to SSRI treatment in depression. Transl Psychiatry 6(11):e957. 10.1038/tp.2016.130 PMC5314109 27874847

[CR39] Gutman AR, Yang Y, Ressler KJ, Davis M (2008) The role of neuropeptide Y in the expression and extinction of fear-potentiated startle. J Neurosci 28(48):12682–12690. 10.1523/JNEUROSCI.2305-08.2008 PMC2621075 19036961

[CR40] Hajos N (2021) Interneuron Types and Their Circuits in the Basolateral Amygdala. Front Neural Circuits 15:687257. 10.3389/fncir.2021.687257 PMC8222668 34177472

[CR41] Heinsen H, Arzberger T, Schmitz C (2000) Celloidin mounting (embedding without infiltration) - a new, simple and reliable method for producing serial sections of high thickness through complete human brains and its application to stereological and immunohistochemical investigations. J Chem Neuroanat 20(1):49–59. 10.1016/s0891-0618(00)00067-3 11074343

[CR42] Hinojosa CA, VanElzakker MB, Hughes KC, Offringa R, Sangermano LM, Spaulding IG, Staples-Bradley LK, Whitman ET, Lasko NB, Rauch SL, Orr SP, Pitman RK, Shin LM (2022) Exaggerated amygdala activation to ambiguous facial expressions is a familial vulnerability factor for posttraumatic stress disorder. J Psychiatr Res 156:451–459. 10.1016/j.jpsychires.2022.10.049 PMC9742331 36332360

[CR43] Holmes A (2008) Genetic variation in cortico-amygdala serotonin function and risk for stress-related disease. Neurosci Biobehav Rev 32(7):1293–1314. 10.1016/j.neubiorev.2008.03.006 PMC2561331 18439676

[CR44] Holmes A, Murphy DL, Crawley JN (2002) Reduced aggression in mice lacking the serotonin transporter. Psychopharmacology 161(2):160–167. 10.1007/s00213-002-1024-3 11981596

[CR46] Holmes A, Murphy DL, Crawley JN (2003b) Abnormal behavioral phenotypes of serotonin transporter knockout mice: parallels with human anxiety and depression. Biol Psychiatry 54(10):953–959. 10.1016/j.biopsych.2003.09.003 14625137

[CR45] Holmes A, Lit Q, Murphy DL, Gold E, Crawley JN (2003a) Abnormal anxiety-related behavior in serotonin transporter null mutant mice: the influence of genetic background. Genes Brain Behav 2(6):365–380. 10.1046/j.1601-1848.2003.00050.x 14653308

[CR47] Homberg JR, Schubert D, Asan E, Aron EN (2016) Sensory processing sensitivity and serotonin gene variance: Insights into mechanisms shaping environmental sensitivity. Neurosci Biobehav Rev 71:472–483. 10.1016/j.neubiorev.2016.09.029 27697602

[CR48] Jansen F, Heiming RS, Lewejohann L, Touma C, Palme R, Schmitt A, Lesch KP, Sachser N (2010) Modulation of behavioural profile and stress response by 5-HTT genotype and social experience in adulthood. Behav Brain Res 207(1):21–29. 10.1016/j.bbr.2009.09.033 19782704

[CR49] Jennings KA, Loder MK, Sheward WJ, Pei Q, Deacon RM, Benson MA, Olverman HJ, Hastie ND, Harmar AJ, Shen S, Sharp T (2006) Increased expression of the 5-HT transporter confers a low-anxiety phenotype linked to decreased 5-HT transmission. J Neurosci 26(35):8955–8964. 10.1523/JNEUROSCI.5356-05.2006 PMC6675351 16943551

[CR50] Kalinowski D, Bogus-Nowakowska K, Kozlowska A, Rowniak M (2023) Dopaminergic and cholinergic modulation of the amygdala is altered in female mice with oestrogen receptor beta deprivation. Sci Rep 13(1):897. 10.1038/s41598-023-28069-2 PMC9845293 36650256

[CR51] Karabeg MM, Grauthoff S, Kollert SY, Weidner M, Heiming RS, Jansen F, Popp S, Kaiser S, Lesch KP, Sachser N, Schmitt AG, Lewejohann L (2013) 5-HTT deficiency affects neuroplasticity and increases stress sensitivity resulting in altered spatial learning performance in the Morris water maze but not in the Barnes maze. PLoS ONE 8(10):e78238. 10.1371/journal.pone.0078238 PMC3805519 24167611

[CR52] Kask A, Harro J, von Horsten S, Redrobe JP, Dumont Y, Quirion R (2002) The neurocircuitry and receptor subtypes mediating anxiolytic-like effects of neuropeptide Y. Neurosci Biobehav Rev 26(3):259–283. 10.1016/s0149-7634(01)00066-5 12034130

[CR53] Kemppainen S, Pitkänen A (2000) Distribution of parvalbumin, calretinin, and calbindin-D(28k) immunoreactivity in the rat amygdaloid complex and colocalization with gamma-aminobutyric acid. J Comp Neurol 426(3):441–467. https://doi.org/10.1002/1096-9861(20001023)426:3<441::aid-cne8>3.0.co;2-7 10.1002/1096-9861(20001023)426:3<441::aid-cne8>3.0.co;2-7 10992249

[CR54] Kim DK, Tolliver TJ, Huang SJ, Martin BJ, Andrews AM, Wichems C, Holmes A, Lesch KP, Murphy DL (2005) Altered serotonin synthesis, turnover and dynamic regulation in multiple brain regions of mice lacking the serotonin transporter. Neuropharmacology 49(6):798–810. 10.1016/j.neuropharm.2005.08.010 16183083

[CR55] Kolter JF, Hildenbrand MF, Popp S, Nauroth S, Bankmann J, Rother L, Waider J, Deckert J, Asan E, Jakob PM, Lesch KP, Schmitt-Bohrer A (2021) Serotonin transporter genotype modulates resting state and predator stress-induced amygdala perfusion in mice in a sex-dependent manner. PLoS ONE 16(2):e0247311. 10.1371/journal.pone.0247311 PMC7895400 33606835

[CR56] Krabbe S, Grundemann J, Luthi A (2018) Amygdala Inhibitory Circuits Regulate Associative Fear Conditioning. Biol Psychiatry 83(10):800–809. 10.1016/j.biopsych.2017.10.006 29174478

[CR57] LeDoux J (2003) The emotional brain, fear, and the amygdala. Cell Mol Neurobiol 23(4–5):727–738. 10.1023/a:1025048802629 PMC11530156 14514027

[CR59] Lesch KP, Waider J (2012) Serotonin in the modulation of neural plasticity and networks: implications for neurodevelopmental disorders. Neuron 76(1):175–191. 10.1016/j.neuron.2012.09.013 23040814

[CR58] Lesch KP, Bengel D, Heils A, Sabol SZ, Greenberg BD, Petri S, Benjamin J, Muller CR, Hamer DH, Murphy DL (1996) Association of anxiety-related traits with a polymorphism in the serotonin transporter gene regulatory region. Science 274(5292):1527–1531. 10.1126/science.274.5292.1527 8929413

[CR60] Lima J, Sharp T, Bannerman DM, McHugh SB (2019) Enhanced discriminative aversive learning and amygdala responsivity in 5-HT transporter mutant mice. Transl Psychiatry 9(1):139. 10.1038/s41398-019-0476-8 PMC6470159 30996249

[CR61] Line SJ, Barkus C, Coyle C, Jennings KA, Deacon RM, Lesch KP, Sharp T, Bannerman DM (2011) Opposing alterations in anxiety and species-typical behaviours in serotonin transporter overexpressor and knockout mice. Eur Neuropsychopharmacol 21(1):108–116. 10.1016/j.euroneuro.2010.08.005 PMC3038260 20863670

[CR63] Mackay JP, Bompolaki M, DeJoseph MR, Michaelson SD, Urban JH, Colmers WF (2019) NPY(2) Receptors Reduce Tonic Action Potential-Independent GABA(B) Currents in the Basolateral Amygdala. J Neurosci 39(25):4909–4930. 10.1523/JNEUROSCI.2226-18.2019 PMC6670257 30971438

[CR64] Manko M, Bienvenu TC, Dalezios Y, Capogna M (2012) Neurogliaform cells of amygdala: a source of slow phasic inhibition in the basolateral complex. J Physiol 590(22):5611–5627. 10.1113/jphysiol.2012.236745 PMC3528981 22930272

[CR65] McDonald AJ (1984) Neuronal organization of the lateral and basolateral amygdaloid nuclei in the rat. J Comp Neurol 222(4):589–606. 10.1002/cne.902220410 6199387

[CR66] McDonald AJ (1989) Coexistence of somatostatin with neuropeptide Y, but not with cholecystokinin or vasoactive intestinal peptide, in neurons of the rat amygdala. Brain Res 500(1–2):37–45. 10.1016/0006-8993(89)90297-7 2575006

[CR67] McDonald AJ (2023) Functional neuroanatomy of monoaminergic systems in the basolateral nuclear complex of the amygdala: Neuronal targets, receptors, and circuits. J Neurosci Res 101(9):1409–1432. 10.1002/jnr.25201 PMC10524224 37166098

[CR69] McDonald AJ, Betette RL (2001) Parvalbumin-containing neurons in the rat basolateral amygdala: morphology and co-localization of Calbindin-D(28k). Neuroscience 102(2):413–425. 10.1016/s0306-4522(00)00481-4 11166127

[CR70] McDonald AJ, Mascagni F (2002) Immunohistochemical characterization of somatostatin containing interneurons in the rat basolateral amygdala. Brain Res 943(2):237–244. 10.1016/s0006-8993(02)02650-1 12101046

[CR71] McDonald AJ, Mascagni F (2007) Neuronal localization of 5-HT type 2A receptor immunoreactivity in the rat basolateral amygdala. Neuroscience 146(1):306–320. 10.1016/j.neuroscience.2007.01.047 PMC1941573 17331657

[CR68] McDonald AJ, Pearson JC (1989) Coexistence of GABA and peptide immunoreactivity in non-pyramidal neurons of the basolateral amygdala. Neurosci Lett 100(1–3):53–58. 10.1016/0304-3940(89)90659-9 2569703

[CR73] McDonald AJ, Zaric V (2015) GABAergic somatostatin-immunoreactive neurons in the amygdala project to the entorhinal cortex. Neuroscience 290:227–242 10.1016/j.neuroscience.2015.01.028 PMC4586050 25637800

[CR72] McDonald AJ, Mascagni F, Zaric V (2012) Subpopulations of somatostatin-immunoreactive non-pyramidal neurons in the amygdala and adjacent external capsule project to the basal forebrain: evidence for the existence of GABAergic projection neurons in the cortical nuclei and basolateral nuclear complex. Front Neural Circuits 6:46. 10.3389/fncir.2012.00046 PMC3402756 22837739

[CR74] Menezes EC, Shah R, Laughlin L, Vinod KY, Smiley JF, Cunha C, Balla A, Sershen H, Castellanos FX, Corvelo A, Teixeira CM (2021) Reduced Motivation in Perinatal Fluoxetine-Treated Mice: A Hypodopaminergic Phenotype. J Neurosci 41(12):2723–2732. 10.1523/JNEUROSCI.2608-20.2021 PMC8018732 33536200

[CR75] Milner TA, Wiley RG, Kurucz OS, Prince SR, Pierce JP (1997) Selective changes in hippocampal neuropeptide Y neurons following removal of the cholinergic septal inputs. J Comp Neurol 386(1):46–59. https://www.ncbi.nlm.nih.gov/pubmed/9303524 9303524

[CR76] Money KM, Stanwood GD (2013) Developmental origins of brain disorders: roles for dopamine. Front Cell Neurosci 7:260. 10.3389/fncel.2013.00260 PMC3867667 24391541

[CR77] Muller JF, Mascagni F, McDonald AJ (2005) Coupled networks of parvalbumin-immunoreactive interneurons in the rat basolateral amygdala. J Neurosci 25(32):7366–7376. 10.1523/JNEUROSCI.0899-05.2005 PMC6725309 16093387

[CR78] Muller JF, Mascagni F, McDonald AJ (2006) Pyramidal cells of the rat basolateral amygdala: synaptology and innervation by parvalbumin-immunoreactive interneurons. J Comp Neurol 494(4):635–650. 10.1002/cne.20832 PMC2562221 16374802

[CR79] Muller JF, Mascagni F, McDonald AJ (2007a) Postsynaptic targets of somatostatin-containing interneurons in the rat basolateral amygdala. J Comp Neurol 500(3):513–529. 10.1002/cne.21185 17120289

[CR80] Muller JF, Mascagni F, McDonald AJ (2007b) Serotonin-immunoreactive axon terminals innervate pyramidal cells and interneurons in the rat basolateral amygdala. J Comp Neurol 505(3):314–335. 10.1002/cne.21486 17879281

[CR81] Muller JF, Mascagni F, McDonald AJ (2009) Dopaminergic innervation of pyramidal cells in the rat basolateral amygdala. Brain Struct Funct 213(3):275–288. 10.1007/s00429-008-0196-y 18839210

[CR82] Murphy DL, Lesch KP (2008) Targeting the murine serotonin transporter: insights into human neurobiology. Nat Rev Neurosci 9(2):85–96. 10.1038/nrn2284 18209729

[CR84] Nguyen PT, Nakamura T, Hori E, Urakawa S, Uwano T, Zhao J, Li R, Bac ND, Hamashima T, Ishii Y, Matsushima T, Ono T, Sasahara M, Nishijo H (2011) Cognitive and socio-emotional deficits in platelet-derived growth factor receptor-beta gene knockout mice. PLoS ONE 6(3):e18004. 10.1371/journal.pone.0018004 PMC3060876 21437241

[CR83] Nietzer SL, Bonn M, Jansen F, Heiming RS, Lewejohann L, Sachser N, Asan ES, Lesch KP, Schmitt AG (2011) Serotonin transporter knockout and repeated social defeat stress: impact on neuronal morphology and plasticity in limbic brain areas. Behav Brain Res 220(1):42–54. 10.1016/j.bbr.2011.01.011 21238500

[CR86] Overstreet-Wadiche L, McBain CJ (2015) Neurogliaform cells in cortical circuits. Nat Rev Neurosci 16(8):458–468. 10.1038/nrn3969 PMC5207343 26189693

[CR85] Özcete OD, Banerjee A, Kaeser PS (2024) Mechanisms of neuromodulatory volume transmission. Mol Psychiatry 29(11):3680–3693. 10.1038/s41380-024-02608-3 PMC11540752 38789677

[CR87] Ozsvar A, Komlosi G, Olah G, Baka J, Molnar G, Tamas G (2021) Predominantly linear summation of metabotropic postsynaptic potentials follows coactivation of neurogliaform interneurons. Elife 10. 10.7554/eLife.65634 PMC8360660 34308838

[CR88] Ozsvar A, Sieburg MC, Sietam MD, Hou WH, Capogna M (2024) A combinatory genetic strategy for targeting neurogliaform neurons in the mouse basolateral amygdala. Front Cell Neurosci 18:1254460. 10.3389/fncel.2024.1254460 PMC10867116 38362542

[CR89] Pape HC, Pare D (2010) Plastic synaptic networks of the amygdala for the acquisition, expression, and extinction of conditioned fear. Physiol Rev 90(2):419–463. 10.1152/physrev.00037.2009 PMC2856122 20393190

[CR90] Pereira PA, Tavares M, Laires M, Mota B, Madeira MD, Paula-Barbosa MM, Cardoso A (2024) Effects of Aging and Nerve Growth Factor on Neuropeptide Expression and Cholinergic Innervation of the Rat Basolateral Amygdala. Biology (Basel) 13(3). 10.3390/biology13030155 PMC10968405 38534426

[CR91] Perrenoud Q, Geoffroy H, Gauthier B, Rancillac A, Alfonsi F, Kessaris N, Rossier J, Vitalis T, Gallopin T (2012) Characterization of Type I and Type II nNOS-Expressing Interneurons in the Barrel Cortex of Mouse. Front Neural Circuits 6:36. 10.3389/fncir.2012.00036 PMC3386492 22754499

[CR92] Pinard CR, Muller JF, Mascagni F, McDonald AJ (2008) Dopaminergic innervation of interneurons in the rat basolateral amygdala. Neuroscience 157(4):850–863. 10.1016/j.neuroscience.2008.09.043 PMC2621110 18948174

[CR93] Pitkänen A, Amaral DG (1993) Distribution of parvalbumin-immunoreactive cells and fibers in the monkey temporal lobe: the hippocampal formation. J Comp Neurol 331(1):37–74. 10.1002/cne.903310104 8320348

[CR94] Pitkänen A, Kemppainen S (2002) Comparison of the distribution of calcium-binding proteins and intrinsic connectivity in the lateral nucleus of the rat, monkey, and human amygdala. Pharmacol Biochem Behav 71(3):369–377. 10.1016/s0091-3057(01)00690-6 11830171

[CR95] Prager EM, Bergstrom HC, Wynn GH, Braga MF (2016) The basolateral amygdala gamma-aminobutyric acidergic system in health and disease. J Neurosci Res 94(6):548–567. 10.1002/jnr.23690 PMC4837071 26586374

[CR96] Rainnie DG, Mania I, Mascagni F, McDonald AJ (2006) Physiological and morphological characterization of parvalbumin-containing interneurons of the rat basolateral amygdala. J Comp Neurol 498(1):142–161. 10.1002/cne.21049 16856165

[CR97] Real MA, Heredia R, Labrador Mdel C, Davila JC, Guirado S (2009) Expression of somatostatin and neuropeptide Y in the embryonic, postnatal, and adult mouse amygdalar complex. J Comp Neurol 513(4):335–348. 10.1002/cne.21970 19177517

[CR99] Rowniak M, Kolenkiewicz M, Kozlowska A (2017) Parvalbumin, but not calretinin, neurons express high levels of alpha1-containing GABA(A) receptors, alpha7-containing nicotinic acetylcholine receptors and D2-dopamine receptors in the basolateral amygdala of the rat. J Chem Neuroanat 86:41–51. 10.1016/j.jchemneu.2017.08.002 28834708

[CR100] Ruijter JM, Ramakers C, Hoogaars WM, Karlen Y, Bakker O, van den Hoff MJ, Moorman AF (2009) Amplification efficiency: linking baseline and bias in the analysis of quantitative PCR data. Nucleic Acids Res 37(6):e45. 10.1093/nar/gkp045 PMC2665230 19237396

[CR101] Sah P, Faber ES, De Armentia L, M., Power J (2003) The amygdaloid complex: anatomy and physiology. Physiol Rev 83(3):803–834. 10.1152/physrev.00002.2003 12843409

[CR102] Sajdyk TJ, Schober DA, Smiley DL, Gehlert DR (2002) Neuropeptide Y-Y2 receptors mediate anxiety in the amygdala. Pharmacol Biochem Behav 71(3):419–423. 10.1016/s0091-3057(01)00679-7 11830176

[CR103] Scheich B, Gaszner B, Kormos V, Laszlo K, Adori C, Borbely E, Hajna Z, Tekus V, Bolcskei K, Abraham I, Pinter E, Szolcsanyi J, Helyes Z (2016) Somatostatin receptor subtype 4 activation is involved in anxiety and depression-like behavior in mouse models. Neuropharmacology 101:204–215. 10.1016/j.neuropharm.2015.09.021 26387439

[CR104] Schwert H (2017) Nicht-pyramidale Neurone als Zielstrukturen des serotonergen und dopaminergen Systems in der basloateralen Amygdala: Untersuchungen an Wildtyp-Mäusen und einem Maus-Modell für emotionale Dysregulation. Würzburg, University of Würzburg

[CR105] Selten M, van Bokhoven H, Nadif Kasri N (2018) Inhibitory control of the excitatory/inhibitory balance in psychiatric disorders. F1000Res 7:23. 10.12688/f1000research.12155.1 PMC5760969 29375819

[CR106] Shackman AJ, Stockbridge MD, Tillman RM, Kaplan CM, Tromp DP, Fox AS, Gamer M (2016) The neurobiology of dispositional negativity and attentional biases to threat: Implications for understanding anxiety disorders in adults and youth. J Exp Psychopathol 7(3):311–342. 10.5127/jep.054015 PMC5130287 27917284

[CR107] Spampanato J, Polepalli J, Sah P (2011) Interneurons in the basolateral amygdala. Neuropharmacology 60(5):765–773. 10.1016/j.neuropharm.2010.11.006 21093462

[CR108] Sperk G (2026) Neuropeptide systems in temporal lobe epilepsy. Neuropeptides 117:102606. 10.1016/j.npep.2026.102606 41825101

[CR109] Stujenske JM, O’Neill PK, Fernandes-Henriques C, Nahmoud I, Goldburg SR, Singh A, Diaz L, Labkovich M, Hardin W, Bolkan SS, Reardon TR, Spellman TJ, Salzman CD, Gordon JA, Liston C, Likhtik E (2022) Prelimbic cortex drives discrimination of non-aversion via amygdala somatostatin interneurons. Neuron 110(14):2258–2267e2211. 10.1016/j.neuron.2022.03.020 PMC9308671 35397211

[CR110] Suri D, Teixeira CM, Cagliostro MK, Mahadevia D, Ansorge MS (2015) Monoamine-sensitive developmental periods impacting adult emotional and cognitive behaviors. Neuropsychopharmacology 40(1):88–112. 10.1038/npp.2014.231 PMC4262911 25178408

[CR111] Takagi H, Somogyi P, Somogyi J, Smith AD (1983) Fine structural studies on a type of somatostatin-immunoreactive neuron and its synaptic connections in the rat neostriatum: a correlated light and electron microscopic study. J Comp Neurol 214(1):1–16. 10.1002/cne.902140102 6132937

[CR112] Tasan RO, Verma D, Wood J, Lach G, Hormer B, de Lima TC, Herzog H, Sperk G (2016) The role of Neuropeptide Y in fear conditioning and extinction. Neuropeptides 55:111–126. 10.1016/j.npep.2015.09.007 26444585

[CR113] Theisen CC, Reyes BAS, Sabban EL, Van Bockstaele EJ (2018) Ultrastructural Characterization of Corticotropin-Releasing Factor and Neuropeptide Y in the Rat Locus Coeruleus: Anatomical Evidence for Putative Interactions. Neuroscience 384:21–40. 10.1016/j.neuroscience.2018.04.043 29775703

[CR114] Tovote P, Fadok JP, Luthi A (2015) Neuronal circuits for fear and anxiety. Nat Rev Neurosci 16(6):317–331. 10.1038/nrn3945 25991441

[CR115] Truitt WA, Johnson PL, Dietrich AD, Fitz SD, Shekhar A (2009) Anxiety-like behavior is modulated by a discrete subpopulation of interneurons in the basolateral amygdala. Neuroscience 160(2):284–294. 10.1016/j.neuroscience.2009.01.083 PMC2682359 19258024

[CR116] Vereczki VK, Veres JM, Muller K, Nagy GA, Racz B, Barsy B, Hajos N (2016) Synaptic Organization of Perisomatic GABAergic Inputs onto the Principal Cells of the Mouse Basolateral Amygdala. Front Neuroanat 10:20. 10.3389/fnana.2016.00020 PMC4779893 27013983

[CR117] Vereczki VK, Muller K, Krizsan E, Mate Z, Fekete Z, Rovira-Esteban L, Veres JM, Erdelyi F, Hajos N (2021) Total Number and Ratio of GABAergic Neuron Types in the Mouse Lateral and Basal Amygdala. J Neurosci 41(21):4575–4595. 10.1523/JNEUROSCI.2700-20.2021 PMC8260245 33837051

[CR118] Wellman CL, Izquierdo A, Garrett JE, Martin KP, Carroll J, Millstein R, Lesch KP, Murphy DL, Holmes A (2007) Impaired stress-coping and fear extinction and abnormal corticolimbic morphology in serotonin transporter knock-out mice. J Neurosci 27(3):684–691. 10.1523/JNEUROSCI.4595-06.2007 PMC6672805 17234600

[CR119] Wolff SB, Grundemann J, Tovote P, Krabbe S, Jacobson GA, Muller C, Herry C, Ehrlich I, Friedrich RW, Letzkus JJ, Luthi A (2014) Amygdala interneuron subtypes control fear learning through disinhibition. Nature 509(7501):453–458. 10.1038/nature13258 24814341

[CR120] Wu YJ, Gu X, Kong Y, Yang S, Wang H, Xu M, Wang Q, Yi X, Lin ZJ, Jiao ZH, Cheung H, Zhao XY, Bian X, Jiang Q, Li Y, Zhu MX, Wang LY, Li Y, Huang J, Xu TL (2026) Neuropeptide Y co-opts neuronal ensembles for memory lability and stability. Nat Neurosci 29(5):1145–1156. 10.1038/s41593-026-02235-x 41917464

[CR121] Xu P, Huang S, Zhang H, Mao C, Zhou XE, Cheng X, Simon IA, Shen DD, Yen HY, Robinson CV, Harpsoe K, Svensson B, Guo J, Jiang H, Gloriam DE, Melcher K, Jiang Y, Zhang Y, Xu HE (2021) Structural insights into the lipid and ligand regulation of serotonin receptors. Nature 592(7854):469–473. 10.1038/s41586-021-03376-8 33762731

[CR122] Yamada S, Kojima K, Tanaka M (2025) Neuropeptide Y neurons in the basolateral amygdala project to the nucleus accumbens and stimulate high-fat intake. Front Cell Neurosci 19:1565939. 10.3389/fncel.2025.1565939 PMC11983651 40213497

[CR123] Yilmazer-Hanke DM, Faber-Zuschratter H, Linke R, Schwegler H (2002) Contribution of amygdala neurons containing peptides and calcium-binding proteins to fear-potentiated startle and exploration-related anxiety in inbred Roman high- and low-avoidance rats. Eur J Neurosci 15(7):1206–1218. 10.1046/j.1460-9568.2002.01945.x 11982631

[CR124] Yilmazer-Hanke DM, Hantsch M, Hanke J, Schulz C, Faber-Zuschratter H, Schwegler H (2004) Neonatal thyroxine treatment: changes in the number of corticotropin-releasing-factor (CRF) and neuropeptide Y (NPY) containing neurons and density of tyrosine hydroxylase positive fibers (TH) in the amygdala correlate with anxiety-related behavior of wistar rats. Neuroscience 124(2):283–297. 10.1016/j.neuroscience.2003.12.004 14980379

[CR125] Yu Q, Teixeira CM, Mahadevia D, Huang Y, Balsam D, Mann JJ, Gingrich JA, Ansorge MS (2014) Dopamine and serotonin signaling during two sensitive developmental periods differentially impact adult aggressive and affective behaviors in mice. Mol Psychiatry 19(6):688–698. 10.1038/mp.2014.10 PMC4311886 24589889

[CR126] Zaborszky L, Heimer L (1989) Cominations of tracer techniques, especially HRP and PHA-L, with transmitter identification for correlated light and electron microscopic studies. In: Zaborszky H (ed) Neuroanatomical tract-tracing methods 2. recent progress. Plenum, New York, pp 49–96. 10.1007/978-1-4757-2055-6_4

[CR127] Zhang ZJ, Lappi DA, Wrenn CC, Milner TA, Wiley RG (1998) Selective lesion of the cholinergic basal forebrain causes a loss of cortical neuropeptide Y and somatostatin neurons. Brain Res 800(2):198–206. 10.1016/s0006-8993(98)00484-3 9685641

[CR128] Zhou Z, Zhu G, Hariri AR, Enoch MA, Scott D, Sinha R, Virkkunen M, Mash DC, Lipsky RH, Hu XZ, Hodgkinson CA, Xu K, Buzas B, Yuan Q, Shen PH, Ferrell RE, Manuck SB, Brown SM, Hauger RL, Goldman D (2008) Genetic variation in human NPY expression affects stress response and emotion. Nature 452(7190):997–1001. 10.1038/nature06858 PMC2715959 18385673

[CR129] Zoli M, Jansson A, Sykova E, Agnati LF, Fuxe K (1999) Volume transmission in the CNS and its relevance for neuropsychopharmacology. Trends Pharmacol Sci 20(4):142–150. 10.1016/s0165-6147(99)01343-7 10322499

