# Supplementary material for: Impact of serotonin transporter deficiency on parvalbumin- and neuropeptide Y-producing neurons of the lateral and basolateral amygdalar nuclei
Source: J Neural Transm (Vienna). 2026 Aug 19;133(9):2635–60. doi: 10.1007/s00702-026-03224-x (PMC13687174; doi:10.1007/s00702-026-03224-x)
Supplement: Supplementary file 1 [file 702_2026_3224_MOESM1_ESM.docx]

**Online Resource 1**

Primer targets and their specifications**.** F: Forward primer sequence; R: Reverse primer sequence. Manufactured by Sigma Aldrich at 100 nM. bp: base pairs.

| Gene | Symbol | Product [bp] | Intron spanning | Accession number/Reference sequence | Primer sequence |
| --- | --- | --- | --- | --- | --- |
| Reference genes | | | | | |
| Actin beta | *Actb* | 84 | no | NM_007393.5 | F: ATGTGGATCAGCAAGCAGGA  R: AGCTCAGTAACAGTCCGCCTA |
| Beta 2 microglobulin | *B2m* | 126 | yes | NM_009735.3 | F: ACCGTCTACTGGGATCGAGA  R: TGCTATTTCTTTCTGCGTGCAT |
| Glyceraldehyde 3-phosphate dehydrogenase | *Gapdh* | 135 | no | NM_008084 | F: GTGATGGGTGTGAACCACGA  R: GGTCATGAGCCCTTCCACAA |
| GDP dissociation inhibitor beta | *Gdi2* | 126 | no | NM_008112 | F: GTCAGAATTGGTTGGTTCTGTTC  R: AGCTCTTGGATCACACAATCG |
| 60S acidic ribosomal protein P0 | *Rplp0* | 83 | yes | NM_007475 | F: GAGGCCACACTGCTGAACAT  R: ATGCTGCCGTTGTCAAACAC |
| Target genes | | | | | |
| Serotonin 1A receptor | *5Htr1a* | 144 | yes | NM_008308.4 | F: GATCTCGCTCACTTGGCTCA  R: AAAGCGCCGAAAGTGGAGTA |
| Serotonin 2A receptor | *5Htr2a* | 112 | yes | NM_172812.3 | F: CCATAGCCGCTTCAACTCCA  R: CGAATCATCCTGTAGCCCGA |
| Serotonin 2C receptor | *5Htr2c* | 73 | yes | NM_008312 | F: GCAATAATGGTGAACCTGGGC  R:ACTGCCAAACCAATAGGCCA |
| Corticotropin-releasing factor | *Crf* | 76 | no | NM_205769 | F: CAACCTCAGCCGGTTCTGAT  R: CAGCGGGACTTCTGTTGAGA |
| Corticotropin-releasing factor receptor 1 | *Crfr1* | 136 | yes | NM_007762 | F: GGAACCTCATCTCGGCTTTCA  R: GTTACGTGGAAGTAGTTGTAGGC |
| Corticotropin-releasing factor receptor 2 | *Crfr2* | 62 | yes | NM_001288618 | F: ATGACGAAGTTACGAGCATCCA  R: GCCTTCACTGCCTTCCTGTATT |
| Neuropeptide Y | *Npy* | 106 | yes | NM_023456 | F: CAGATACTACTCCGCTCTGCGACACTACAT  R: TTCCTTCATTAAGAGGTCTGAAATCAGTGTCT |
| Neuropeptide Y receptor 1 | *Npyr1* | 105 | yes | NM_010934 | F: ATTTCGGCCCACTCTGCTTT  R: ACCTGTACTTACTGTCCCGGA |
| Neuropeptide Y receptor 2 | *Npyr2* | 77 | yes | NM_001205099 | F: CATCTGAGAAGGAACGCGCA  R: CTACCGGGCCCATCTTCAGA |
| Somatostatin | *Som* | 112 | yes | NM_009215 | F: GACCCCAGACTCCGTCAGTTT  R: TCTCTGTCTGGTTGGGCTCG |
| Somatostatin receptor type 2 | *Sstr2* | 97 | no | NM_001042606 | F: GAGCAGTTGAATGGGAGCCA  R: AGTATGGCTCGGTCTGGTTG |
| Somatostatin receptor type 4 | *Sstr4* | 92 | no | NM_009219 | F: AGCGGGCATGGTCACTATC  R: AGCGTAGGATCACGAAGATGA |

**Online resource 2**

Table 2.1. Descriptive and explorative statistics of quantitative analysis of PV-ir cells in the amygdala of 5-HTT wild-type (WT) and knockout (KO) mice. Shown are the data (Mean ± SEM) for volume, number, and density of PV-ir cells in different (sub)-nuclei of the amygdala. Statistical analysis was performed using mixed two-way ANOVA followed by the Bonferroni’s multiple comparison test as a post hoc-test. BL, basolateral nucleus; BLa, anterior part of the BL; BLp, posterior part of the BL; La, lateral nucleus.

| **Mean±SEM, group size (n) Mixed two-way ANOVA** | | | | | | | | | | | | |
| --- | --- | --- | --- | --- | --- | --- | --- | --- | --- | --- | --- | --- |
|  | BL ges. WT | BL ges. KO | La ges. WT | La ges. KO |  | Region |  | Genotype |  | Interaction |  | Bonferroni’s multiple comparison test |
|  |  |  |  |  |  | F(1.15) | p | F(1.15) | p | F(1.15) | p |  |
| *Volume* | 60.99 ± 3.27 (8) | 63.21 ± 1.49 (8) | 30.56 ± 3.67 (9) | 37.97 ± 2.54 (8) |  | 390.701 | **< .001** | 1.608 | .225 | 3.386 | 0.087 |  |
| *Number* | 191.38 ± 15.81 (8) | 177.88 ± 18.19 (8) | 43.75 ± 7.64 (8) | 42.88 ± 6.52 (8) |  | 259.583 | **< .001** | 0.196 | .665 | 0.518 | .484 |  |
| *Density* | 3.11 ± 0.12 (8) | 2.81 ± 0.27 (8) | 1.43 ± 0.23(8) | 1.14 ± 0.16 (8) |  | 193.978 | **< .001** | 1.285 | .276 | 0.002 | .969 |  |
|  | BLa_WT | BLa_KO | BLp_WT | BLp_KO |  | Region |  | Genotype |  | Interaction |  | Bonferroni’s multiple comparison test |
|  |  |  |  |  |  | F(1.11) | p | F(1.11) | p | F(1.11) | p |  |
| *Volume* | 48.48 ± 2.22 (5) | 50.72 ± 1.69 (8) | 10.32 ± 2.04 (5) | 12.49 ± 1.16 (8) |  | 420.111 | **< .001** | 1.810 | .206 | 0.000 | .983 |  |
| *Number* | 143.00 ± 12.67 (5) | 121.25 ± 12.44 (8) | 49.00 ± 11.02 (5) | 56.63 ± 6.60 (8) |  | 139.661 | **< .001** | 0.246 | 0.629 | 4.789 | .051 |  |
| *Density* | 2.93 ± 0.13 (5) | 2.40 ± 0.23 (8) | 4.64 ± 0.20 (5) | 4.56 ± 0.40 (8) |  | 117.499 | **< .001** | 0.557 | 0.471 | 1.538 | .241 |  |
|  | | | | |  |  | | | | | | |

Table 2.2. Descriptive and exploratory statistics for the quantitative analysis of PV-ir cells in the basolateral amygdala (BLA) as well as in the lateral (La) and basolateral (BL) nuclei of the amygdala in 5-HTT wild-type (WT) and knockout (KO) mice along the rostral-caudal axis. Shown are the data (Mean ± SEM) for volume, number, and density of PV-ir cells in the rostral, intermediate (interm.), and caudal part of the BLA, BL, or La. Statistical analysis was performed using mixed two-way ANOVA followed by the Bonferroni’s multiple comparison test as a post hoc-test.

| **Mean±SEM, group size (n)** | | | | | | |  | **Mixed two-way ANOVA** | | | | | | |  |
| --- | --- | --- | --- | --- | --- | --- | --- | --- | --- | --- | --- | --- | --- | --- | --- |
|  | BLA rostral _WT | BLA rostral _KO | BLA interm. _WT | BLA interm. _KO | BLA caudal _WT | BLA caudal _KO |  | Region |  | Geno-type |  | Interaction |  | Bonferroni’s multiple comparison test | |
|  |  |  |  |  |  |  |  | F(2.12) | p | F(2.12) | p | F(2.12) | p |  | |
| *Volume* | 14.04 ± 1.64 (7) | 15.62 ± 0.62 (8) | 34.66 ± 4.08 (7) | 40.78 ± 1.28 (8) | 47.00 ± 1.84 (7) | 48.22 ± 2.72 (8) |  | 201.722 | **< .001** | 1.372 | .262 | 1.328 | .282 | Regardless of *5-Htt* genotype:  Rostral vs. intermediate: **p < .001**; rostral vs. caudal: **p < .001**; intermediate vs. caudal: **p < .001** | |
| *Number* | 28.72 ± 7.00 (7) | 25.62 ± 3.44 (8) | 101.58 ± 11.04 (7) | 98.62 ± 12.12 (8) | 119.72 ± 11.6 (7) | 102.74 ± 8.72 (8) |  | 83.212 | **< .001** | 0.529 | .480 | 0.647 | .532 | Regardless of *5-Htt* genotype:  Rostral vs. intermediate: **p < .001**; rostral vs. caudal: **p < .001**; intermediate vs. caudal: p = .447 | |
| *Density* | 1.88 ± 0.31 (7) | 1.56 ± 0.26 (8) | 2.93 ± 0.17 (7) | 2.39 ± 0.27 (7) | 2.58 ± 0.28 (7) | 2.21 ± 0.24 (8) |  | 10.821 | **< .001** | 2.230 | .159 | 0.159 | .854 | Regardless of *5-Htt* genotype:  Rostral vs. intermediate: **p < .001**; rostral vs. caudal: p = .057; intermediate vs. caudal: p = .490 | |
|  | BL rostral _WT | BL rostral _KO | BL interm. _WT | BL interm. _KO | BL caudal _WT | BL caudal _KO |  | Region |  | Geno-type |  | Interaction |  | Bonferroni’s multiple comparison test | |
|  | | | | | | |  | F(2.12) | p | F(2.12) | p | F(2.12) | p |  | |
| *Volume* | 10.86 ± 1.22 (7) | 11.56 ± 0.50 (8) | 24.54 ± 2.0 (7) | 26.82 ± 0.96 (8) | 28.04 ± 1.46 (7) | 26.56 ± 0.82 (8) |  | 119.433 | **< .001** | 0.211 | .653 | 1.358 | .275 | Regardless of *5-Htt* genotype:  Rostral vs. intermediate: **p < .001**; rostral vs. caudal: **p < .001**; intermediate vs. caudal: p = .771 | |
| *Number* | 24.58 ± 6.0 (7) | 21.38 ± 2.96 (8) | 82.72 ± 6.10 (7) | 79.38 ± 10.56 (8) | 88.86 ± 7.10 (7) | 80.50 ± 6.12 (8) |  | 94.576 | **< .001** | 0.394 | .541 | 0.170 | .821 | Regardless of *5-Htt* genotype:  Rostral vs. intermediate: **p < .001**; rostral vs. caudal: **p < .001**; intermediate vs. caudal: p = 1.0 | |
| *Density* | 2.12 ± 0.43 (7) | 1.75 ± 0.26 (8) | 3.4 ± 0.22 (7) | 2.92 ± 0.38 (7) | 3.23 ± 0.29 (7) | 3.11 ± 0.26 (8) |  | 14.877 | **< .001** | 0.998 | .336 | 0.256 | .776 | Regardless of *5-Htt* genotype:  Rostral vs. intermediate: **p = .002**; rostral vs. caudal: **p = .004**; intermediate vs. caudal: p = 1.0 | |
|  | La rostral _WT | La rostral _KO | La interm. _WT | La interm. _KO | La caudal _WT | La caudal _KO |  | Region |  | Geno-type |  | Interaction |  | Bonferroni’s multiple comparison test | |
|  | | | | | | |  | F(2.12) | p | F(2.12) | p | F(2.12) | p |  | |
| *Volume* | 3.18 ± 0.86 (7) | 4.04 ± 0.30 (8) | 10.12 ± 2.22 (7) | 13.96 ± 0.56 (8) | 18.96 ± 1.48 (7) | 21.66 ± 2.10 (8) |  | 114.298 | **< .001** | 2.426 | .143 | 0.917 | ,412 | Regardless of *5-Htt* genotype:  Rostral vs. intermediate: **p < .001**; rostral vs. caudal: **p < .001**; intermediate vs. caudal: **p < .001** | |
| *Number* | 4.14 ± 1.18 (7) | 4.26 ± 0.74 (8) | 18.86 ± 8.26 (7) | 19.26 ± 3.28 (8) | 30.86 ± 6.64 (7) | 22.26 ± 3.28 (8) |  | 12.812 | **< .001** | .508 | .488 | 0.647 | .532 | Regardless of *5-Htt* genotype:  Rostral vs. intermediate: **p = .006**; rostral vs. caudal: **p < .001**; intermediate vs. caudal: p = .602 | |
| *Density* | 1.22 ± 0.28 (7) | 1.00 ± 0.29 (8) | 1.48 ± 0.31 (7) | 1.31 ± 0.21 (7) | 1.62 ± 0.30 (7) | 1.10 ± 0.19 (8) |  | 0.698 | .506 | 2.017 | .179 | 0.266 | .769 |  | |
|  | | | | | | |  |  | | | | | | | |

**Online Resource 3**

Table 3.1. Descriptive and explorative statistics of quantitative analysis of NPY-ir cells in the amygdala of 5-HTT wild-type (WT) and knockout (KO) mice. Shown are the data (Mean ± SEM) for volume, number, and density of NPY-ir cells in different (sub)-nuclei of the amygdala as well as the number and density of round and fusiform NPY-ir cells, the two types of NPY-ir cells found in the basolateral amygdala (BLA). Statistical analysis was performed using mixed two-way ANOVA followed by the Bonferroni’s multiple comparison test as a post hoc-test. Solely for the statistical comparison of the BLA volume between WT and KO mice an independent samples t-test was applied. BL, basolateral nucleus; BLa, anterior part of the BL; BLp, posterior part of the BL; La, lateral nucleus.

| **Mean±SEM, group size (n)** | | | | |  | **Mixed two-way ANOVA** | | | | | | |  |
| --- | --- | --- | --- | --- | --- | --- | --- | --- | --- | --- | --- | --- | --- |
|  | BL _WT | BL_KO | La _WT | La_KO |  | Region |  | Genotype |  | Interaction |  | Bonferroni’s multiple comparison test | |
|  |  |  |  |  |  | F(1.15) | p | F(1.15) | p | F(1.15) | p |  | |
| *Volume* | 73,24 ± 4.45 (9) | 75.85 ± 3.24 (8) | 35.34 ± 2.52 (9) | 38.45 ± 2.19 (8) |  | 427.698 | **< .001** | 0.448 | .513 | 0.019 | .892 |  | |
| *Number* | 94.22 ± 5.71 (9) | 54.25 ± 4.81 (8) | 33.22 ± 2.41 (9) | 21.37 ± 1.75 (8) |  | 175.455 | **< .001** | 32.345 | **< .001** | 15.749 | **.001** | In both WT and KO: BL vs. La **p < .001**;  BL WT vs. BL KO: **p < .001**;  La WT vs. La KO: **p = .001** | |
| *Density* | 1.31 ± 0.08 (9) | 0.73 ± 0.09 (8) | 0.98 ± 0.1 (9) | 0.58 ± 0.073 (8) |  | 21.499 | **< .001** | 18.370 | **< .001** | 2.956 | .106 |  | |
|  | BLa_WT | BLa_KO | BLp_WT | BLp_KO |  | Region |  | Genotype |  | Interaction |  | Bonferroni’s multiple comparison test | |
|  |  |  |  |  |  | F(1.14) | p | F(1.14) | p | F(1.14) | p |  | |
| *Volume* | 59.04 ± 2.92 (8) | 64.55 ± 2.45 (8) | 15.66 ± 2.47 (8) | 11.30 ± 1.39 (8) |  | 788.589 | **< .001** | 0.040 | .844 | 8.235 | .012 |  | |
| *Number* | 79 ± 6.33 (8) | 49.88 ± 4.69 (8) | 17.00 ± 1.50 (8) | 4.38 ± 0.96 (8) |  | 166.791 | **< .001** | 28.596 | **< .001** | 3.929 | .067 |  | |
| *Density* | 1.35 ± 0.10 (8) | 0.79 ± 0.10 (8) | 1.18 ± 0.11 (8) | 0.38 ± 0.07 (8) |  | 15.307 | **0.002** | 34.058 | **< .001** | 2.560 | .132 |  | |
|  | BLA_ round_WT | BLA round_KO | BLA fusiform_WT | BLA fusiform_KO |  | Region |  | Genotype |  | Interaction |  | Bonferroni’s multiple comparison test | |
|  |  |  |  |  |  | F(1.15) | p | F(1.15) | p | F(1.15) | p |  | |
| *Volume* | BLA_WT:  108.58 ± 6.80 (9) | BLA_KO:  114.30 ± 4.83 (8) |  |  |  | t-test for independent samples: p (two-tailed) = 0.513 | | | | | | | |
| *Number* | 71.11 ± 3.57 (9) | 47.50 ± 3.20 (8) | 56.33 ± 5.09 (9) | 28.13 ± 2.74 (8) |  | 32.480 | **< .001** | 32.345 | **< .001** | 0.589 | .455 |  | |
| *Density* | .67 ± 0.04 (9) | 0.43 ± 0.05 (8) | 0.53 ± 0.05(9) | 0.25 ± 0.03 (8) |  | 27.858 | **< .001** | 20.514 | **< .001** | 0.412 | .531 |  | |

Table 3.2. Descriptive and exploratory statistics for the quantitative analysis of NPY-ir cells in the basolateral amygdala (BLA) as well as in the lateral (La) and basolateral (BL) nuclei of the amygdala in 5-HTT wild-type (WT) and knockout (KO) mice along the rostral-caudal axis. Shown are the data (Mean ± SEM) for volume, number, and density of NPY-ir cells in rostral, intermediate (interm.), and caudal part of the BLA. Statistical analysis was performed using mixed two-way ANOVA followed by the Bonferroni’s multiple comparison test as a post hoc-test.

| **Mean±SEM, group size (n)** | | | | | | | | |  | **Mixed two-way ANOVA** | | | | | | |
| --- | --- | --- | --- | --- | --- | --- | --- | --- | --- | --- | --- | --- | --- | --- | --- | --- |
|  | BLA rostral _WT | BLA rostral _KO | BLA interm. _WT | BLA interm. _KO | | BLA caudal _WT | | BLA caudal _KO |  | Region |  | Geno-type |  | Interaction |  | Bonferroni’s multiple comparison test |
|  |  |  |  |  | | | | |  | F(2.13) | p | F(2.13) | p | F(2.13) | p |  |
| *Volume* | 16.96 ± 1.82 (9) | 21.72 ± 1.32 (7) | 40.32 ± 3.16 (9) | 45.16 ± 2.44 (7) | | 51.28 ± 3.04 (9) | | 50.50 ± 2.44 (7) |  | 142.186 | **< .001** | 1.048 | .323 | 1.372 | .269 | Regardless of *5-Htt* genotype:  Rostral vs. intermediate: **p < .001**; rostral vs. caudal: **p < .001**; intermediate vs. caudal: **p = .009** |
| *Number* | 36.44 ± 3.16 (9) | 22.58 ± 2.40 (7) | 45.78 ± 4.06 (9) | 31.58 ± 1.98 (7) | | 45.22 ± 2.52 (9) | | 16.58 ± 1.62 (7) |  | 7.085 | **.003** | 32.345 | **< .001** | 5.166 | **.012** | WT vs. KO in BLA rostral **p = 0.005**, in BLA interm. **p = 0.012**, in BLA caudal **p < .001**  In WT: BLA rostral vs. intermed. p = 0.052; BLA rostral vs. caudal. p = 0.069; BLA interm. vs. caudal p = 1.0  In KO: BLA rostral vs. interm. p = 0.11; BLA rostral vs. caudal. p = 0.44; BLA interm. vs. BLA caudal **p = 0.006** |
| *Density* | 2.34 ± 0.12 (9) | 1.17 ± 0.11 (7) | 1.18 ± 0.12 (9) | 0.72 ± 0.05 (7) | | 0.90 ± 0.07 (9) | | 0.34 ± 0.03 (7) |  | 113.917 | **< .001** | 48.618 | **< .001** | 12.162 | **< .001** | WT vs. KO in BLA rostral **p < .001**, in BLA interm. **p = 0.005**, in BLA caudal **p < .001;**  In WT: BLA rostral vs. intermed. **p < .001**; BLA rostral vs. caudal. **p < .001**; BLA interm. vs. caudal **p = .017**  In KO: BLA rostral vs. interm. **p = 0.014**; BLA rostral vs. caudal. **p < .001**; BLA interm. vs. BLA caudal **p = 0.004** |
|  | BL rostral_WT | BL rostral_KO | BL interm._WT | BL interm._KO | BL caudal_WT | | BL caudal_KO | |  | Region |  | Geno-type |  | Interaction |  | Bonferroni’s multiple comparison test |
|  | | | | | | | | |  | F(2.13) | p | F(2.13) | p | F(2.13) | p |  |
| *Volume* | 13.78 ± 1.32 (9) | 16.94 ± 0.96 (7) | 28.00 ± 1.38 (9) | 30.28 ± 1.78 (7) | | 31.46 ± 2.82 (9) | | 30.88 ± 0.86 (7) |  | 74.153 | **< .001** | 0.733 | .406 | ,960 | .364 | Regardless of *5-Htt* genotype:  Rostral vs. intermediate: **p < .001**; rostral vs. caudal: **p < .001**; intermediate vs. caudal: p = .663 |
| *Number* | 28.00 ± 3.12 (9) | 20.42 ± 3.72 (7) | 32.00 ± 2.68 (9) | 21.42 ± 2.04 (7) | | 31.00 ± 1.78 (9) | | 11.14 ± 1.76 (7) |  | 4.519 | **0.020** | 16.857 | **.001** | 5.797 | **.008** | WT vs. KO in BL rostral p = .141, in BL interm. **p = 0.010**, in BL caudal **p < .001;** In WT: BL rostral vs. intermed. p = 0.509; BL rostral vs. caudal. p = .752; BL interm. vs. caudal p = 1.0  In KO: BL rostral vs. interm**.** p = 1.0; BL rostral vs. caudal. **p = 0.017**; BL interm. vs. BLA caudal **p = 0.003** |
| *Density* | 2.26 ± 0.11 (9) | 1.41 ± 0.32 (7) | 1.15 ± 0.09 (9) | 0.73 ± 0.06 (7) | | 1.05 ± 0.09 (9) | | 0.37 ± 0.06 (7) |  | 48.616 | **< .001** | 21.089 | **< .001** | 1.506 | .243 | In both WT and KO together: rostral vs. interm. **p < .001**; rostral vs. caudal. **p < .001**; interm. vs. caudal **p = .007.** |
|  | La rostral_WT | La rostral_KO | La interm._WT | La interm._KO | | La caudal_WT | | La caudal_KO |  | Region |  | Geno-type |  | Interaction |  | Bonferroni’s multiple comparison test |
|  | | | | | | | | |  | F(2.13) | p | F(2.13) | p | F(2.13) | p |  |
| Volume | 3.76 ± 0.58 (9) | 4.74 ± 0.44 (7) | 12.80 ± 1.90 (9) | 14.98 ± 0.96 (7) | | 18.76 ± 1.42 (9) | | 20.84 ± 2.06 (7) |  | 62.474 | **< .001** | 2.278 | .153 | 0.110 | .896 | Regardless of *5-Htt* genotype:  Rostral vs. intermediate: **p < .001**; rostral vs. caudal: **p < .001**; intermediate vs. caudal: **p = .013** |
| Number | 8.44 ± 1.32 (9) | 7.14 ± 1.84 (7) | 13.78 ± 1.76 (9) | 9.58 ± 1.72 (7) | | 14.22 ± 2.94 (9) | | 7.86 ± 2.26 (7) |  | 1.836 | .178 | 6.318 | **.025** | .684 | .513 |  |
| Density | 2.58 ± 0.48 (9) | 1.82 ± 0.42 (7) | 1.20 ± 0.24 (9) | 0.69 ± 0.14 (7) | | 0.649 ± 0.30 (9) | | 0.36 ± 0.08 (7) |  | 17.631 | **< .001** | 2.684 | .124 | 0.317 | .731 | In both WT and KO together: rostral vs. interm. **p = .003**; rostral vs. caudal. **p < .001**; interm. vs. caudal p = .198. |
|  | | | | | | | | |  |  | | | | | | |

**Online Resource 4**

Descriptive and explorative statistics of quantitative real-time PCR data. Expression levels in the BLA of 5-HTT WT and 5-HTT KO mice are shown. Pairwise comparison was performed using either the parametric T-test or the non-parametric Mann-Whitney U-test. Significant p-values are indicated by bold numbers.

| Gene | Mean (± SEM) of different genotype | | Levene’s test  (new)  p-value | Shapiro-Wilk Test  p-value | | T-test  p-value | Mann Whitney U- test  p-value |
| --- | --- | --- | --- | --- | --- | --- | --- |
|  | 5-HTT WT  (n=10) | 5-HTT KO  (n=8) |  | 5-HTT WT | 5-HTT KO |  |  |
| *5Htr1a* | 1.03  (± 0.16) | 1.12  (± 0.09) | 0.354 | **0.001** | **0.025** |  | 0.101 |
| *5Htr2a* | 0.99  (± 0.05) | 1.05  (± 0.07) | 0.366 | 0.909 | 0.607 | 0.444 |  |
| *5Htr2c* | 0.91  (± 0.08) | 1.11  (± 0.06) | 0.251 | 0.311 | 0.764 | 0.077 |  |
| *Crf* | 0.99  (± 0.11) | 1.28  (± 0.24) | **0.026** | 0.594 | 0.229 |  | 0.408 |
| *Crfr1* | 0.88  (± 0.03) | 1.26  (± 0.09) | 0.077 | 0.323 | **0.049** |  | **<0.001** |
| *Crfr2* | 0.90  (± 0.06) | 1.26  (± 0.15) | **0.011** | 0.687 | 0.517 |  | 0.055 |
| *Npy* | 0.99  (± 0.03) | 0.97  (± 0.04) | 0.239 | 0.420 | 0.631 | 0.932 |  |
| *Npyr1* | 0.97  (± 0.04) | 1.12  (± 0.07) | 0.314 | 0. 125 | 0. 943 | 0.079 |  |
| *Npyr2* | 0.97  (± 0.05) | 1.16  (± 0.06) | 0.965 | 0.221 | 0.438 | **0.036** |  |
| *Som* | 1.04  (± 0.06) | 1.01  (± 0.04) | 0.137 | 0.122 | 0.569 | 0.706 |  |
| *Sstr2* | 0.92  (± 0.09) | 1.18  (± 0.12) | 0.571 | 0.096 | 0.506 | 0.102 |  |
| *Sstr4* | 0.91  (± 0.04) | 1.16  (± 0.07) | 0.245 | 0.916 | 0.912 | **0.006** |  |
